# Two distinct conformations of factor H regulate discrete complement-binding functions in the fluid phase and at cell surfaces

**DOI:** 10.1074/jbc.RA118.004767

**Published:** 2018-09-14

**Authors:** Amy J. Osborne, Ruodan Nan, Ami Miller, Jayesh S. Bhatt, Jayesh Gor, Stephen J. Perkins

**Affiliations:** From the Department of Structural and Molecular Biology, Darwin Building, University College London, Gower Street, London WC1E 6BT, United Kingdom

**Keywords:** C3b activity, immune system, innate immunity, solution structure, analytical ultracentrifugation, complement, molecular modeling, surface plasmon resonance (SPR), X-ray scattering, complement regulation, Monte Carlo modeling

## Abstract

Factor H (FH) is the major regulator of C3b in the alternative pathway of the complement system in immunity. FH comprises 20 short complement regulator (SCR) domains, including eight glycans, and its Y402H polymorphism predisposes those who carry it to age-related macular degeneration. To better understand FH complement binding and self-association, we have studied the solution structures of both the His-402 and Tyr-402 FH allotypes. Analytical ultracentrifugation revealed that up to 12% of both FH allotypes self-associate, and this was confirmed by small-angle X-ray scattering (SAXS), MS, and surface plasmon resonance analyses. SAXS showed that monomeric FH has a radius of gyration (*R_g_*) of 7.2–7.8 nm and a length of 25 nm. Starting from known structures for the SCR domains and glycans, the SAXS data were fitted using Monte Carlo methods to determine atomistic structures of monomeric FH. The analysis of 29,715 physically realistic but randomized FH conformations resulted in 100 similar best-fit FH structures for each allotype. Two distinct molecular structures resulted that showed either an extended N-terminal domain arrangement with a folded-back C terminus or an extended C terminus and a folded-back N terminus. These two structures are the most accurate to date for glycosylated full-length FH. To clarify FH functional roles in host protection, crystal structures for the FH complexes with C3b and C3dg revealed that the extended N-terminal conformation accounted for C3b fluid-phase regulation, the extended C-terminal conformation accounted for C3d binding, and both conformations accounted for bivalent FH binding to glycosaminoglycans on the target cell surface.

## Introduction

The alternative pathway of complement is activated by the spontaneous hydrolysis of C3 into C3u, also known as C3(H_2_O). This leads to a positive-feedback amplification of C3 cleavage to form activated C3b that opsonizes pathogenic cells. To prevent unwanted C3b-mediated host cell damage, complement factor H (FH)^3^ regulates complement by acting as a cofactor for factor I (FI) to cleave C3b (1–3), competing with factor B (FB) to interfere with the formation of the C3 convertase C3bBb (4), and accelerating the decay of the C3bBb complex (2, 5). These activities occur in the fluid phase and less effectively at the host cell surface (6).

Disrupted complement regulation is associated with age-related macular degeneration (AMD), the most common cause of blindness in the West (7–10), and also with atypical hemolytic uremic syndrome (aHUS), C3 glomerulopathy (C3G), and Alzheimer's disease (11–13). In early stage AMD, sub-retinal pigment epithelial deposits known as drusen develop within Bruch's membrane, which is an extracellular matrix layer interposed between the retinal pigment epithelium and the choroidal vasculature (14). Drusen contains oxidized lipids, carbohydrates, cellular materials, and over 200 proteins, including FH and other complement components (10, 15, 16).

**Figure 1.**
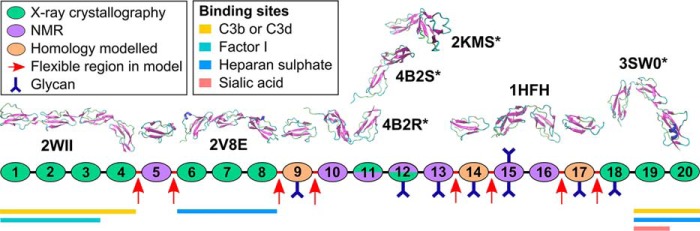
**Cartoon of the 20 SCR domains in FH.** Each SCR domain is represented by an *ellipse* color-coded to indicate the starting atomistic structure (*green,* X-ray crystallography; *purple,* NMR; *orange,* homology modeling). Ribbon views of the seven SCR structures used to model FH are shown above the *cartoon*, together with their PDB codes. The *asterisks* signify newer SCR structural models that became available after the previous full-length FH models were published. Below the *cartoon*, the FH functional binding sites are denoted by *horizontal bars* for each of C3b, C3d, factor I, heparan sulfate, and sialic acid.

FH is composed of 20 short complement regulator (SCR) domains of ∼61 residues connected by linkers of 3–8 residues in length (Fig. 1) (17). Eight out of nine potential *N*-glycosylation sites in FH are occupied by biantennary disialylated glycans (18). Functionally, FH has multiple binding sites for its major ligands C3b, the C3d fragment, C-reactive protein (CRP), and two host-cell markers heparin, which is an analogue of the natural ligand heparan sulfate (HS), and sialic acid (19–21). Each ligand binds to FH weakly with micromolar *K_D_* values (22) at specific SCR domains summarized in Fig. 1. For example, FH has two binding sites for heparin at SCR-7 and SCR-20 (22–26), and weak independent interactions with heparin at SCR-7 and SCR-20 result in bivalent binding with an overall greater affinity than either site on their own (21, 27). The dimerization of FH at high concentrations was first reported by X-ray and neutron scattering (28). FH also self-associates at each of SCR-6/8 and SCR-16/20 with micromolar *K_D_* values; the sequential daisy-chaining of these two dimer sites leads to FH oligomers (26, 29–31). The formation of dimers and tetramers of FH may also be involved in host cell recognition essential for complement regulation (32). However, it is often presumed that FH is monomeric (33).

Genetic variants in FH are associated with disease (34). In AMD, FH SCR-7 harbors the AMD-risk polymorphism Y402H (7–10). Individuals homozygous for His-402 have a 6-fold increased risk of developing AMD compared with 2.5-fold for His-402 heterozygotes (35). The presence of His-402 weakened heparin binding to both FH SCR-6/8 and full-length FH (36, 37), FH binding to CRP (38, 39), and full-length FH binding to HS in Bruch's membrane (40). For the development of AMD, age-related changes in glycosaminoglycan structures such as a decrease in the sulfation of HS, together with the presence of FH His-402, have been proposed to reduce FH binding over time. At host cell surfaces, FH His-402 showed weaker SCR-7 binding to eye tissue-specific HS only (41). In opposition to these observations of weaker HS binding for the His-402 allotype, His-402 in SCR-6/8 was seen to be bound to the highly-sulfated glycosaminoglycan analogue sucrose octasulfate using X-ray crystallography, whereas no such crystals were reported for the Tyr-402 allotype (42). Although the statistical association of Y402H with AMD is one of the strongest, many other complement variants in *CFH* and other complement genes associate also with AMD, suggesting that AMD is caused by several different molecular mechanisms. For example, at the other heparin-binding site on FH, SCR-20 harbors the AMD-risk rare variant R1210C (43). AMD is associated with another 12 rare variants in FH in its signal peptide or in SCR-1, SCR-3/5, SCR-8, SCR-16, or SCR-18 (44). AMD is also associated with the protective polymorphism V62I in SCR-1 (10, 45), common synonymous and noncoding variants in a region overlapping the *CFH* gene (36, 46, 47), and genetic variants in complement *C3, CFI, CFB*, and *C9* (44, 49).

Additional insights into molecular mechanisms for AMD may be gained by studying both the self-association and overall solution structure of FH in the context of the Y402H polymorphism. To date, full-length FH has proven too large, flexible, and glycosylated for high-resolution structural determination by crystallography or NMR. Preliminary molecular modeling of small angle X-ray scattering (SAXS) curves revealed that full-length FH in solution has a folded-back SCR structure that is affected by ionic strength (26, 50, 51). To examine the effect of the Y402H polymorphism on FH self-association and its solution structure, we submitted each of the homozygous FH Tyr-402 and His-402 to analytical ultracentrifugation (AUC), SAXS, MS, and surface plasmon resonance studies. Both FH allotypes self-associated to form oligomers, and SAXS curve extrapolation to zero concentration showed that both FH allotypes exhibited similar overall structures. Our preliminary FH modeling was based on just 11–14 high-resolution SCR structures and an initial molecular dynamics approach to model the inter-SCR linkers (26, 51). Here, the FH modeling is much improved by starting from 17 high-resolution SCR structures (Fig. 1; Table 1) (26, 45, 52, 53) and utilizing a much improved Monte Carlo atomistic modeling method for the full-length FH structure, including the eight FH glycan chains (54). The resulting SAXS best-fit structural ensembles corresponded to the most accurate FH models determined to date. These ensembles revealed two different extended and folded-back FH domain arrangements that favor two different modes of FH binding to either C3b or C3d. Both arrangements accounted for the bivalent binding of FH to HS-coated cell surfaces. The outcome of two different FH conformations provides new insights into the way in which FH regulates complement C3b activity.

**Table 1:** **Sources of molecular SCR structures in FH**

FH domain(s)	PDB code	Ref.	Method	Used in previous FH model
SCR-1/4	2WII	52	X-ray crystallography	51
SCR-1/3	2RLP/2RLQ	45	NMR	26
SCR-4		112	Homology	26
SCR-5		95	NMR	26, 51
SCR-6/8	2V8E	113	X-ray crystallography	26, 51
SCR-9*^a^*		112	Homology	26, 51
SCR-10/11*^b^*	4B2R	114	NMR	
SCR-10/13		112	Homology	26
SCR-11/12*^b^*	4B2S	114	X-ray crystallography	
SCR-12/13	2KMS	53	NMR	51
SCR-14*^a^*		112	Homology	26, 51
SCR-15/16	1HFH	115	NMR	26, 51
SCR-17*^a^*		112	Homology	26, 51
SCR-18		112	Homology	26
SCR-18/20*^b^*	3SW0	79	X-ray crystallography	
SCR-19/20	2G7I	48	X-ray crystallography	26, 51

*^a^* Only homology models are available for this (Fig. 1).

*^b^* Newer SCR structural models published after the previous full-length FH models were published (Fig. 1).

## Results

### Sedimentation velocity analyses of the two FH allotypes

**Figure 2.**
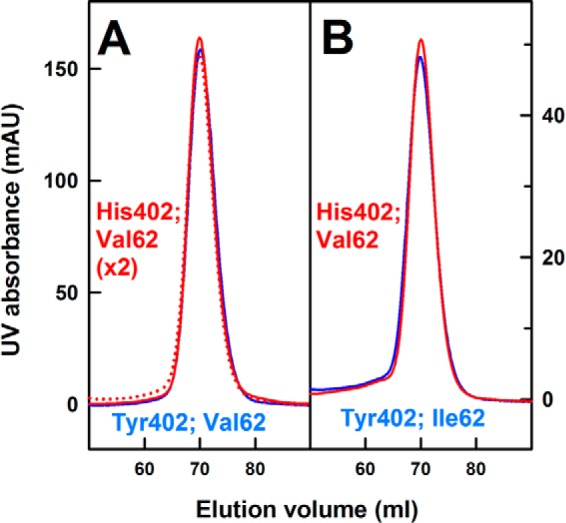
**Size-exclusion gel filtration of homozygous FH.** Pairs of homozygous purified FH Tyr-402 and His-402 at *A*, 0.9 mg/ml, and *B*, 0.3 mg/ml, were loaded onto a Superose^TM^ 6 prep grade XK 16/60 column immediately after AUC sedimentation velocity experiments. Here and below, data for the Tyr-402 and His-402 allotypes are denoted in *blue* and *red*, respectively.

AUC studies use macromolecular sedimentation behavior under a high centrifugal force to determine sizes and shapes (55). The full-length FH Tyr-402 and FH His-402 allotypes were purified from the plasma of genotyped volunteers (Table 3) as single peaks from size-exclusion chromatography (Fig. 2) that gave clean single bands by SDS-PAGE (29). Here and below, FH was genotyped for both Y402H and I62V polymorphisms, with our focus on the former, whereas the presence of Val-62 or Ile-62 in the FH samples was noted for completion but was not investigated further (56). Previous fluid-phase activity assays using C3u and FI with both FH allotypes showed that both demonstrated similar C3u cleavage rates (57).

**Figure 3.**
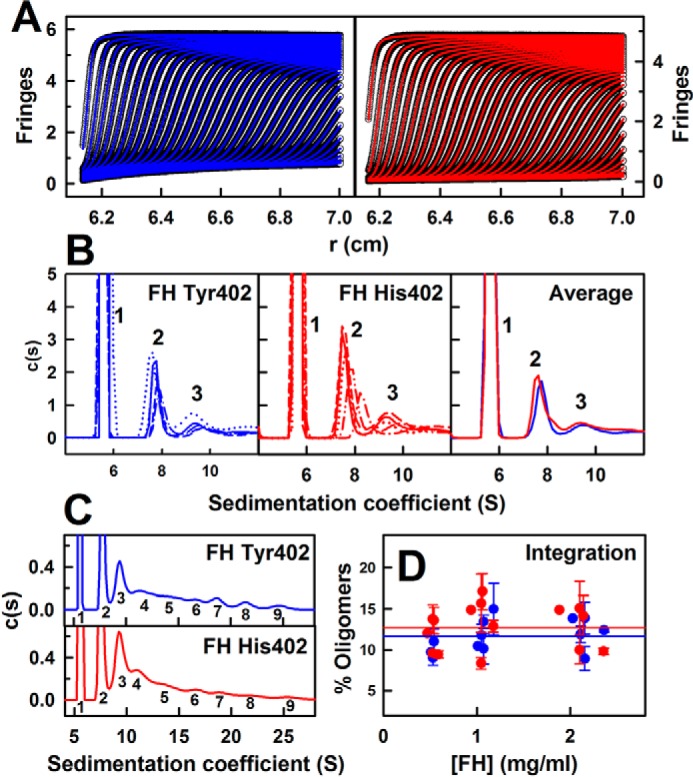
**Sedimentation velocity *c*(*s*) size-distribution analyses for FH Tyr-402 and FH His-402.** All analyses correspond to 1.1 mg/ml FH. *A*, representative sedimentation boundary fits corresponding to FH Tyr-402 (*blue*) and FH His-402 (*red*) are shown using only every 10th scan for clarity. The experimental data are shown in *black. B*, peaks 1–3 in the *c*(*s*) analyses for five FH Tyr-402 and five FH His-402 samples (*red*) are shown in different *line styles*. The averaged *c*(*s*) plots for FH Tyr-402 and FH His-402 are shown at the *right*. The monomer peak 1 at 5.7 S is normalized to 100. *C*, full *c*(*s*) analyses for FH Tyr-402 and FH His-402 reveal up to nine FH oligomer species starting with peaks 1 and 2 for monomer and dimer and extending to peak 9 for presumed nonamers. *D*, proportion of FH oligomers was derived by integration of the *c*(*s*) analyses for peaks 2–9 for the five FH Tyr-402 and FH His-402 samples at concentrations between 0.5 and 2.4 mg/ml. Statistical *error bars* are shown where visible. The mean proportion of oligomers is shown as *horizontal lines*.

The higher oligomers previously observed for heterozygous FH (26, 29–31) were reassessed using AUC sedimentation velocity experiments on five homozygous FH Tyr-402 and five homozygous FH His-402/Val-62 samples at three concentrations between 0.5 and 2.1 mg/ml in HEPES buffers (10 mm HEPES, 137 mm NaCl, pH 7.4). Similar sedimentation boundaries were observed for the two allotypes (Fig. 3*A*), and these were fitted using size-distribution analyses *c*(*s*). Good fits with low residuals were obtained in all 10 cases. The mean sedimentation coefficient *s*_20,_ *_w_* of monomeric FH (Tyr-402) at 50,000 rpm was 5.69 ± 0.03 S and that for FH (His-402) was identical at 5.67 ± 0.04 S (*peak 1* in Fig. 3*B*). Both values (Table 3) agree well with previous *s*_20,_ *_w_* values of 5.3 ± 0.1 S (50) and 5.65 ± 0.05 S (29) for WT monomeric FH in 137 mm NaCl. Conversion of the *c*(*s*) distribution to mass distribution plots *c*(*M*) showed that the major peak 1 corresponded to a molecular mass of 147 ± 10 kDa for FH Tyr-402 and 147 ± 11 kDa for FH His-402. Both values agreed with the sequence-calculated molecular mass of 154 kDa for both allotypes and previous *c*(*M*) mass determinations of 142 ± 2 kDa for native FH (29). Integration showed that the dimer and trimer peaks comprised 4.0 ± 1.9 and 2.1 ± 0.7%, respectively, for FH Tyr-402 and 5.7 ± 1.5 and 2.5 ± 0.6%, respectively, for FH His-402. Averaging the *c*(*s*) distributions established the observation of these dimer and trimer peaks (Fig. 3*B*). Hence, at 1.1 mg/ml, the His-402 allotype formed slightly more dimer and trimer than the Tyr-402 allotype, but the difference was small (*peaks 2* and *3* in Fig. 3*B*). The mean *s*_20,_ *_w_* value of dimeric FH Tyr-402 was 7.57 ± 0.36 S, and that for dimeric FH His-402 was similar at 7.69 ± 0.40 S (*peak 2* in Fig. 3*B*). The mean *s*_20,_ *_w_* value of trimeric FH Tyr-402 was 9.3 ± 0.7 S and that for trimeric FH His-402 was similar at 9.4 ± 0.8 S (*peak 3* in Fig. 3*B*). These values agree well with previous values of 7.3 ± 0.2 and 9.2 ± 0.2 S for heterozygous WT FH dimers and trimers, respectively, in a range of NaCl concentrations and pH values, and their size increases with the increase in FH concentration (26, 29). In general, reaction boundaries give single *c*(*s*) peaks that correspond to the co-sedimentation of two different species in rapid equilibrium and exhibit well-defined sedimentation coefficients between the values of the two different species (58). The observation of distinct dimer and trimer *c*(*s*) peaks thus corresponded to slow equilibria between monomer, dimer, and trimer FH on the time scale of sedimentation.

AUC had showed previously that FH was described by a reversible monomer–dimer equilibrium up to 1.3 mg/ml and that heterozygous FH above 2 mg/ml forms irreversible high oligomers (29). To assess these with homozygous FH, size-exclusion chromatography was performed after the proteins had sedimented by AUC. After resuspending by gentle shaking, size-exclusion chromatography showed that the single homogeneous FH peak between 65 and 75 ml was supplemented by small additional peaks corresponding to irreversible FH oligomers that eluted earlier between 55 and 65 ml in all cases, as expected (Fig. 2). The main monomer peak was eluted at the same position in each case, indicating no structural differences between the allotypes.

In heterozygous WT FH, oligomers from tetramers to nonamers have previously been observed at concentrations as low as 0.4 mg/ml (26, 29). Here, their existence was also observed for FH Tyr-402 at 1.1 mg/ml, and the *s*_20,_ *_w_* values of peaks 4–9 (Fig. 3*C*) were 11.9 ± 1.0 S, 14.7 ± 0.8 S, 17.1 ± 0.7 S, 19.4 ± 1.0 S, 22.1 ± 1.0 S, and 25.0 ± 0.7 S in that order. For FH His-402 at 1.1 mg/ml, the *s*_20,_ *_w_* values of peaks 4–9 (Fig. 3*C*) were in agreement at 11.9 ± 0.9 S, 14.3 ± 0.5 S, 16.7 ± 0.4 S, 18.7 ± 0.6 S, 21.3 ± 0.5 S, and 24.8 ± 0.7 S in that order. Integrations of peaks 2–9 yielded the percentages of oligomers (Fig. 3*D*). Values of 11.7 ± 2.0 and 12.8 ± 2.7% of oligomers were seen for FH Tyr-402 and FH His-402, respectively, with this being marginally higher for FH His-402. Both percentages are consistent with an estimate of 17% of dimers predicted from a self-association *K_D_* value of 28 μm (29).

### Mass spectrometry of the two FH allotypes

**Figure 4.**
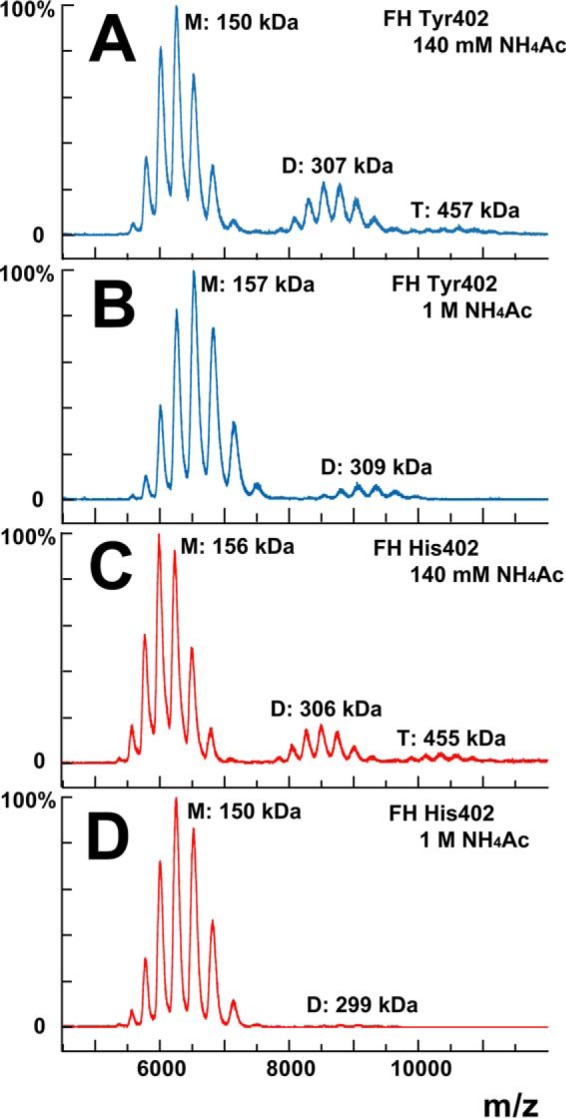
**Mass spectrometry of FH Tyr-402 and FH His-402.** The FH monomer, dimer, and trimer peaks are marked by *M*, *D*, and *T* and their measured molecular weights. *A*, homozygous FH Tyr-402/Val-62 was studied at 3.9 mg/ml (25.3 μm) in 140 mm ammonium acetate buffer, pH 7.0. *B*, same FH Tyr-402/Val-62 sample was studied at 4.4 mg/ml (28.6 μm) in 1 m ammonium acetate buffer, pH 7.0. *C*, homozygous FH His-402/Val-62 was studied at 3.9 mg/ml (25.3 μm) in 140 mm ammonium acetate buffer, pH 7.0. *D*, same FH His-402/Val-62 sample was studied at 3.6 mg/ml (23.4 μm) in 1 m ammonium acetate buffer, pH 7.0.

Because FH self-association had only previously been reported by AUC, MS of homozygous FH was used to further examine self-association. MS observes macromolecular assemblies and determines their stoichiometry (59). One pair of homozygous FH Tyr-402/Val-62 and His-402/Val-62 allotypes was studied shortly after their concentration to about 4 mg/ml in order to promote the formation of oligomers. The use of 140 mm ammonium acetate buffer, pH 7.0, and 1 m ammonium acetate buffer, pH 7.0, evaluated two ionic strengths, given the decrease in oligomers with an increase in the NaCl concentration (26). Ammonium acetate was used because this is volatile in the gas phase used for measurements, unlike the HEPES buffer used for AUC. All four samples showed signals with five to six components at mass/charge (*m/z*) ratios that corresponded to the FH monomer with charges of +23 to +27 (Fig. 4). For FH Tyr-402, the monomer mass was determined to be 150 kDa in 140 mm ammonium acetate and 157 kDa in 1 m ammonium acetate (Fig. 4, *A* and *B*). Similar masses were obtained for FH His-402, giving 156 kDa in 140 mm ammonium acetate and 150 kDa in 1 m ammonium acetate (Fig. 4, *C* and *D*). These masses agreed well with those from AUC (26, 29). They also agreed with the sequence-calculated mass of 154 kDa for FH based on eight glycan chains (18). The variability of the mass determinations was attributed to glycan heterogeneity in FH.

Dimer and trimer FH were also observed in the mass spectra (Fig. 4). For both FH Tyr-402 and His-402, the dimer and trimer were seen in 140 mm ammonium acetate, whereas only the dimer was seen in 1 m ammonium acetate. Their molecular masses were determined to be 307 and 457 kDa and 309 kDa, respectively (Fig. 4, *A* and *B*). Similar results were obtained for FH His-402, now giving masses of 306 and 455 kDa in 140 mm ammonium acetate, and 299 kDa in 1 m ammonium acetate (Fig. 4, *C* and *D*). Peak integrations gave the proportions of monomer, dimer, and trimer as 73, 24, and 4% for FH Tyr-402 and 76, 17, and 6% for FH His-402 in 140 mm ammonium acetate (Fig. 4, *A* and *C*). The appearance of dimer and trimer peaks in the mass spectra validates the observation of dimer and trimer by AUC (Fig. 3*B*), and the summed integration was comparable with 11.7–12.8% by AUC from Fig. 3*D*. At 4 mg/ml, dimer formation was estimated as 37% from a *K_D_* value of 28 μm, with this being comparable with the observed amounts of dimer. The proportions of monomer and dimer changed to 90 and 10% for FH Tyr-402 and 98 and 2% for FH His-402 in 1 m ammonium acetate (Fig. 4, *B* and *D*). The reduction of dimer and the disappearance of trimer in 1 m ammonium acetate agrees well with the effect of increased ionic strength in reducing FH self-association (26). No difference was detectable between the two allotypes.

### Surface plasmon resonance of the two FH allotypes and FH SCR-6/8

**Figure 5.**
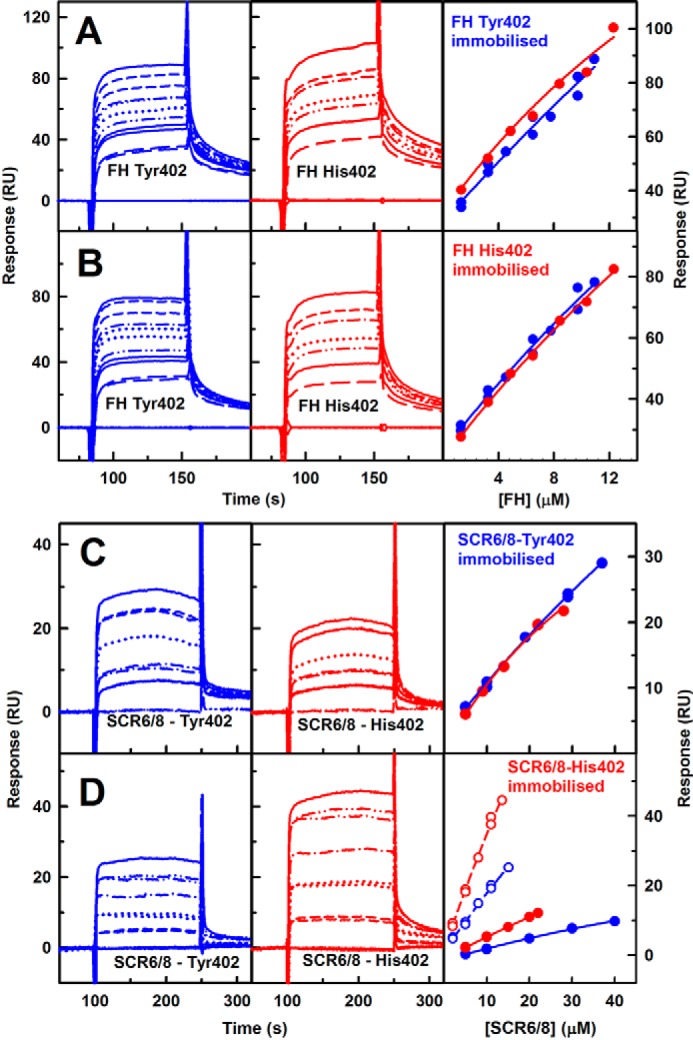
**Surface plasmon resonance analysis of FH Tyr-402 and FH His-402 self-association.** The sensorgrams are shown after subtraction of that recorded with the buffer only. In the *right panels*, no *K_D_* values were determined because these were greater than 40 μm. *A*, 800 RU of homozygous FH Tyr-402/Val-62 was immobilized on a CM3 chip. FH Tyr-402/Val-62 was analyzed at eight concentrations of 0 μm (measured twice), 1.30 μm (twice), 3.24 μm (twice), 4.53 μm, 6.48 μm (twice), 7.77 μm, 9.72 μm (twice), and 10.93 μm from *bottom to top*. FH His-402/Val-62 was analyzed at eight concentrations of 0 μm (twice) and 1.30, 3.24, 4.86, 6.48, 8.42, 10.37, and 12.31 μm. Here and below, binding affinities were fitted using the maximum response values. *B*, 800 RU of homozygous FH His-402/Val-62 was immobilized on a CM3 chip. The same FH Tyr-402/Val-62 and FH His-402/Val-62 concentrations were used. *C*, 150 RU of SCR-6/8 Tyr-402 was immobilized on a CM4 chip. SCR-6/8 Tyr-402 was analyzed at concentrations of six concentrations of 0 μm (twice), 5 and 10 μm (twice), 19 μm, 29 μm (twice), and 37 μm from *bottom to top*. For the His-402 allotype, six concentrations of 0 μm (twice), 5 and 10 μm (twice), 19 and 29 μm (twice), and 37 μm were used, each measured in duplicate. *D*, 400 RU of SCR-6/8 His-402 was immobilized on a CM4 chip The binding affinities were likewise fitted using the maximum response values (*open circles*). A second experiment with 150 RU of SCR-6/8 His-402 immobilized on a CM4 chip and six concentrations of 0, 5, 10, 15, 20, and 22 μm SCR-6/8 is also shown (*filled circles*).

Surface plasmon resonance (SPR) of the binding of solution-phase FH to surface-immobilized FH further enabled FH self-association to be monitored (60). 800 RU of each of homozygous FH Tyr-402 and FH His-402/Val-62 were immobilized on CM3 chips. Equilibrium experiments were performed by flowing FH Tyr-402 over the chip at eight concentrations between 1.3 and 10.9 μm and FH His-402 at concentrations between 1.3 and 12.3 μm in HEPES buffer. The binding of both FH allotypes to their immobilized FH partner was observed at all concentrations (Fig. 5, *A* and *B*) indicating that FH self-association proceeded in physiologically relevant conditions. In equilibrium analyses, the individual sensorgrams could not be fitted to a 1:1 binding kinetic model (data not shown). This key outcome indicated that FH self-association could not be explained as a 1:1 model and that more than one self-association site existed in FH with different affinities, in agreement with the multiple oligomers seen by AUC (29). Alternative models to fit the SPR data were not attempted because of the complication that both the ligand and analyte showed bivalent binding for each other. The maximum binding responses were used to fit a steady-state affinity model. An estimated dissociation constant *K_D_* value of 8.0 μm with a χ^2^ value of 2.6 RU^2^ was determined for FH Tyr-402, which was almost identical to an estimated *K_D_* value of 6.7 μm with a χ^2^ value of 6.5 RU^2^ determined for FH His-402 (Fig. 5, *A* and *B*). Both values were smaller than the *K_D_* value of 28 μm determined for WT FH in solution by sedimentation equilibrium AUC (29). The stronger self-association seen by SPR than by AUC may result from different avidity effects at a surface that amplifies the strength of binding, although the linearity of the binding data in a comparatively low FH concentration range precluded a quantitative comparison with the AUC value.

The SPR results were examined further using the SCR-6/8 fragment. Each SCR-6/8 allotype was immobilized to a CM4 chip up to 150 RU, and the His-402 allotype was also immobilized up to 400 RU on a third chip (Fig. 5, *C* and *D*). The binding of SCR-6/8 Tyr-402 was studied between 0.1 and 0.8 mg/ml (5–40 μm). The binding of SCR-6/8 His-402 was studied between 0.1 and 0.6 mg/ml (5–28 μm). Both allotypes self-associated with each other. The immobilized Tyr-402 allotype showed the same binding affinities to both allotypes (Fig. 5*C*). However, immobilized His-402 allotype showed a higher self-association with His-402 than with Tyr-402 (Fig. 5*D*). This supported earlier AUC experiments suggesting that the His-402 allotype of SCR-6/8 self-associated more than the Tyr-402 allotype (30). The *K_D_* values could not be determined from steady-state affinity fits because the *K_D_* values were weaker than 40 μm; however, this outcome agreed with the *K_D_* value of 40 μm from AUC (30). Comparison of this value with the lower *K_D_* values of 28 μm for FH self-association by AUC (29) and 7–8 μm by SPR (above) showed that other self-association sites must exist in FH as well as in SCR-6/8.

**Figure 6.**
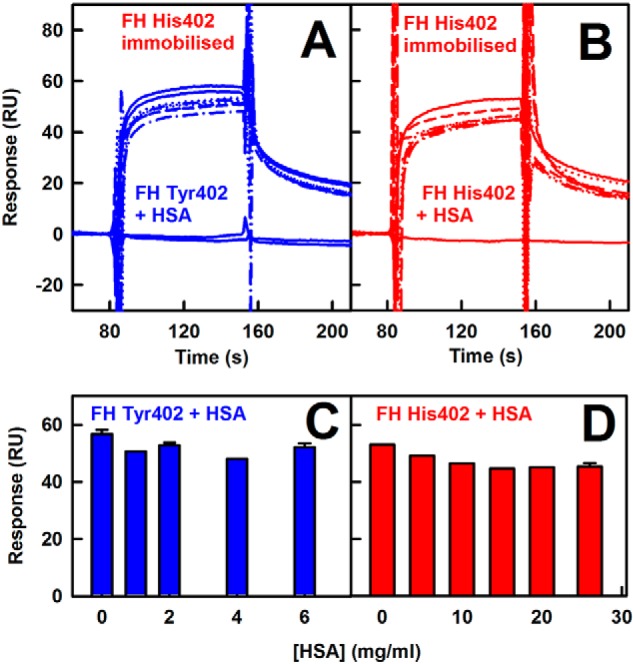
**Surface plasmon resonance analysis of FH self-association in the presence of HSA.** 800 RU of FH His-402 was immobilized on a CM3 chip. The sensorgrams are shown after subtraction of the sensorgram with those of HSA at the corresponding concentrations. The buffer sensorgram is shown at the *bottom. A*, FH Tyr-402/Val-62 at 4.53 μm was analyzed with HSA at concentrations between 0 and 6 mg/ml. *B*, FH His-402 at 4.53 μm was analyzed with HSA at concentrations of 0–26 mg/ml. *C* and *D*, *bar charts* show the maximum responses from *A* and *B*, respectively. *Error bars* are shown where large enough to be observed.

Human serum albumin is the most abundant protein in serum, being present at 35–50 mg/ml. As a control of potential nonspecific protein–protein interactions that might influence FH self-association, further SPR experiments were performed with solution-phase FH Tyr-402 and His-402 flowed over immobilized FH His-402 in the presence of up to 6 and 25 mg/ml human serum albumin (Fig. 6, *A* and *B*). FH self-association continued to be observed even in the presence of high amounts of albumin (Fig. 6, *C* and *D*).

### X-ray scattering of the two FH allotypes

**Figure 7.**
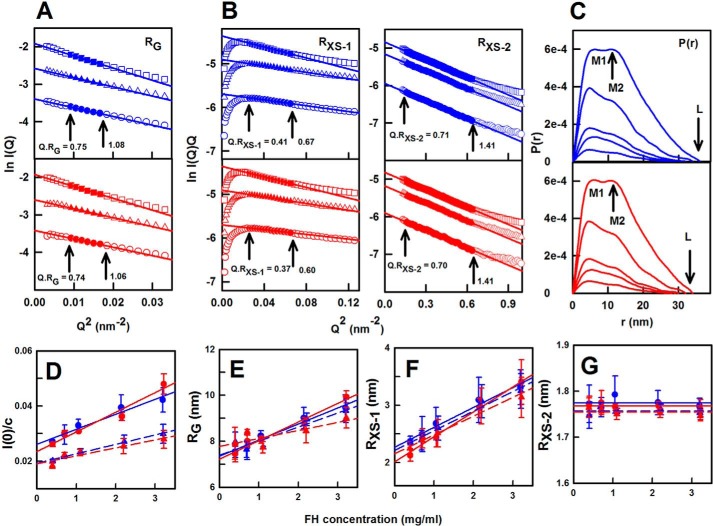
**X-ray scattering analyses of FH Tyr-402 and FH His-402.** In the *R_g_* and *R_XS_* Guinier analyses, the *filled symbols* correspond to the *I*(*Q*) data used to determine the *R_g_* or *R_XS_* values, and the *straight lines* correspond to the best fit through those points. The *Q*·*R_g_* fit ranges are *arrowed. A*, *R_g_* plots of ln *I*(*Q*) *versus Q*^2^ at low *Q* values for FH Tyr-402 (*blue*) and FH His-402/Val-62 (*red*) at concentrations of 1.1 mg/ml (○), 2.2 mg/ml (▵), and 3.2 mg/ml (□). The *Q* fit range was 0.09–0.13 nm^−1^. *B*, corresponding cross-sectional *R_XS_*_-1_ and *R_XS_*_-2_ fits of ln *I*(*Q*). *Q versus Q*^2^ values are shown using *Q* fit ranges of 0.16–0.26 and 0.4–0.8 nm^−1^, respectively. *C*, distance distribution function *P*(*r*) analyses are shown at concentrations of 0.4, 0.7, 1.1, 2.2, and 3.3 mg/ml. *D–G*, concentration dependences of the *I*(0)/*c*, *R_g_, R_XS_*_-1_, and *R_XS_*_-2_ parameters are shown for FH Tyr-402 and FH Tyr-402/Val-62 (*blue*; ● and ▴) and FH His-402/Val-62 (*red*; ● and ▴). Each value was measured in quadruplicate and then averaged and fitted by linear regression except in *G* when the mean was shown. Statistical *error bars* are shown where visible. *D*, *I*(0)/*c* intensities were measured in two beam sessions, resulting in two different pairs of lines. *E*, the *R_g_* values at zero concentration were 7.39 ± 0.25 and 7.35 ± 0.13 nm (FH Tyr-402) and 7.22 ± 0.15 and 7.77 ± 0.27 nm (FH His-402) (Table 3). *F, R_XS_*_-1_ values at zero concentration were 2.21 ± 0.06 and 2.27 ± 0.06 nm (FH Tyr-402), and 2.02 ± 0.06 and 2.15 ± 0.06 nm (FH His-402). *G*, averaged *R_XS_*_-2_ values were 1.77 and 1.77 nm (FH Tyr-402) and 1.76 and 1.75 nm (FH His-402).

X-ray scattering is a diffraction method to determine solution structures of macromolecules in random orientations (61). Here, two pairs of homozygous FH Tyr-402 and FH His-402/Val-62 samples (Table 3) were each freshly prepared and studied in two different sessions in five concentrations between 0.4 mg/ml (2.6 μm) and 3.3 mg/ml (21.4 μm) in HEPES buffer. Excellent signal-to-noise ratios were obtained with no detectable effect from radiation damage. Linear Guinier fits at low *Q* values (where *Q* = 4π sin θ/λ; 2θ = scattering angle; λ = wavelength) gave the radius of gyration (*R_g_*) in satisfactory *Q*·*R_g_* ranges below 1.1 (Fig. 7*A*). The *R_g_* value monitors the degree of elongation of FH. At larger *Q* values, the cross-sectional Guinier *R_XS_* fits monitor the structural proximity between non-neighboring SCR domains (denoted as *R_XS_*_-1_) and between neighboring SCR domains (denoted as *R_XS_*_-2_) (50). Both the *R_XS_*_-1_ and *R_XS_*_-2_ parameters were likewise obtained from linear fits within satisfactory *Q*·*R_XS_* ranges (Fig. 7*B*). This permitted comparative data analyses for the four FH samples. At 0.4 mg/ml, the *R_g_* values were 7.54 and 7.97 nm for both FH Tyr-402/Val-62 samples and were 7.45 and 7.91 nm for both FH His-402/Val-62 samples. The *R_XS_*_-1_ values were 2.35 and 2.36 nm for FH Tyr-402 and 2.15 and 2.25 nm for FH His-402. The *R_XS_*_-2_ values were 1.77 and 1.76 nm for FH Tyr-402 and 1.77 and 1.75 nm for FH His-402. Because the *R_g_*, *R_XS_*_-1_, and *R_XS_*_-2_ parameters at 0.4 mg/ml were similar for both allotypes, no conformational differences between the allotypes were detectable.

Both FH Tyr-402 and FH His-402 showed concentration dependences for *I*(0)/*c*, *R_g_*, and *R_XS_*_-1_ but not for *R_XS_*_-2_ (Fig. 7, *D–G*). These concentration dependences showed self-association for both FH allotypes, the extent of which was similar to WT FH. The *I*(0)/*c* value is proportional to molecular mass. Assuming the slopes in Fig. 7*D* corresponded to a monomer–dimer equilibrium, the *K_D_* values were estimated to be 16 ± 4 and 18 ± 2 μm for the two FH Tyr-402 samples and 11 ± 3 and 22 ± 8 μm for the two FH His-402 samples. These *K_D_* values were comparable with that of 28 μm from AUC (29) and those of 7–8 μm from SPR (Fig. 1). The extrapolation of the *R_g_* values to zero concentration, in order to represent the monomer, gave values of 7.22–7.77 nm (Fig. 7*E*; Table 3). These values were lower than that of 8.90 ± 0.19 nm for WT FH (29); this reduction was attributed to reduced oligomer formation in improved homozygous FH preparations. The lower *R_g_* value of 7.22–7.77 nm indicated that FH was even more folded-back in its SCR domain arrangement than previously thought. The *R_XS_*_-1_ values at zero concentration were 2.02–2.27 nm, which were lower when compared with the previous *R_XS_*_-1_ value of 2.51 ± 0.06 nm (29). The mean *R_XS_*_-2_ value was 1.75–1.77 nm, being similar to the previous *R_XS_*_-2_ value of 1.79 ± 0.01 nm (29).

The distance distribution function *P*(*r*) reports the distances between all pairs of atoms within FH (Fig. 7*C*). The *P*(*r*) curves gave another calculation of the *R_g_* and *I*(0) values for FH Tyr-402 and FH His-402 (Table 3). At 0.4 mg/ml, the Guinier and *P*(*r*) *R_g_* values showed good agreement. The maximum dimension *L* of FH resulted from the intercept of the *P*(*r*) curves with zero at large *r.* The *r* value of the maximum *M* in the *P*(*r*) curve gives the most commonly occurring distance within FH. The two FH allotypes showed similar concentration dependences in their *P*(*r*) curves and *L* values (Fig. 7*C*), indicating similar solution structures for the two allotypes. For FH Tyr-402, a concentration-independent peak *M1* was seen at *r* = 5.3 ± 0.7 nm, and a concentration-dependent second peak *M2* was seen at *r* = 11 nm at 3.3 mg/ml. For FH His-402, *M1* was unchanged at 4.9 ± 0.4 nm, and *M2* was also 11 nm at 3.3 mg/ml. The concentration dependence of *M2* reflected FH oligomer formation. At 0.4 mg/ml, the *L* values were 28–29 nm for FH Tyr-402, and 26–28 nm for FH His-402 (Table 3), but were increased at higher concentrations (Fig. 7*C*). These *L* values were lower than those previously reported (29), and this is attributable to a reduced FH oligomerization at lower concentrations.

In normalized Kratky analyses of (*Q*·*R_g_*)^2^·*I*(*Q*)*/I*(0) *versus Q*·*R_g_* for the four FH experimental curves (62), a clear peak was obtained in all four cases that tailed off at large *Q.R_g_* values (data shown below). These are representative of extended multidomain proteins tethered by linkers of varying conformations. The Tyr-402 and His-402 allotypes showed very similar peaks at *Q*·*R_g_* values of 4.22 and 4.28 respectively, indicating that the two allotypes exhibited similar extended flexible structures.

### FH models for the Tyr-402 and His-402 allotypes

**Figure 8.**
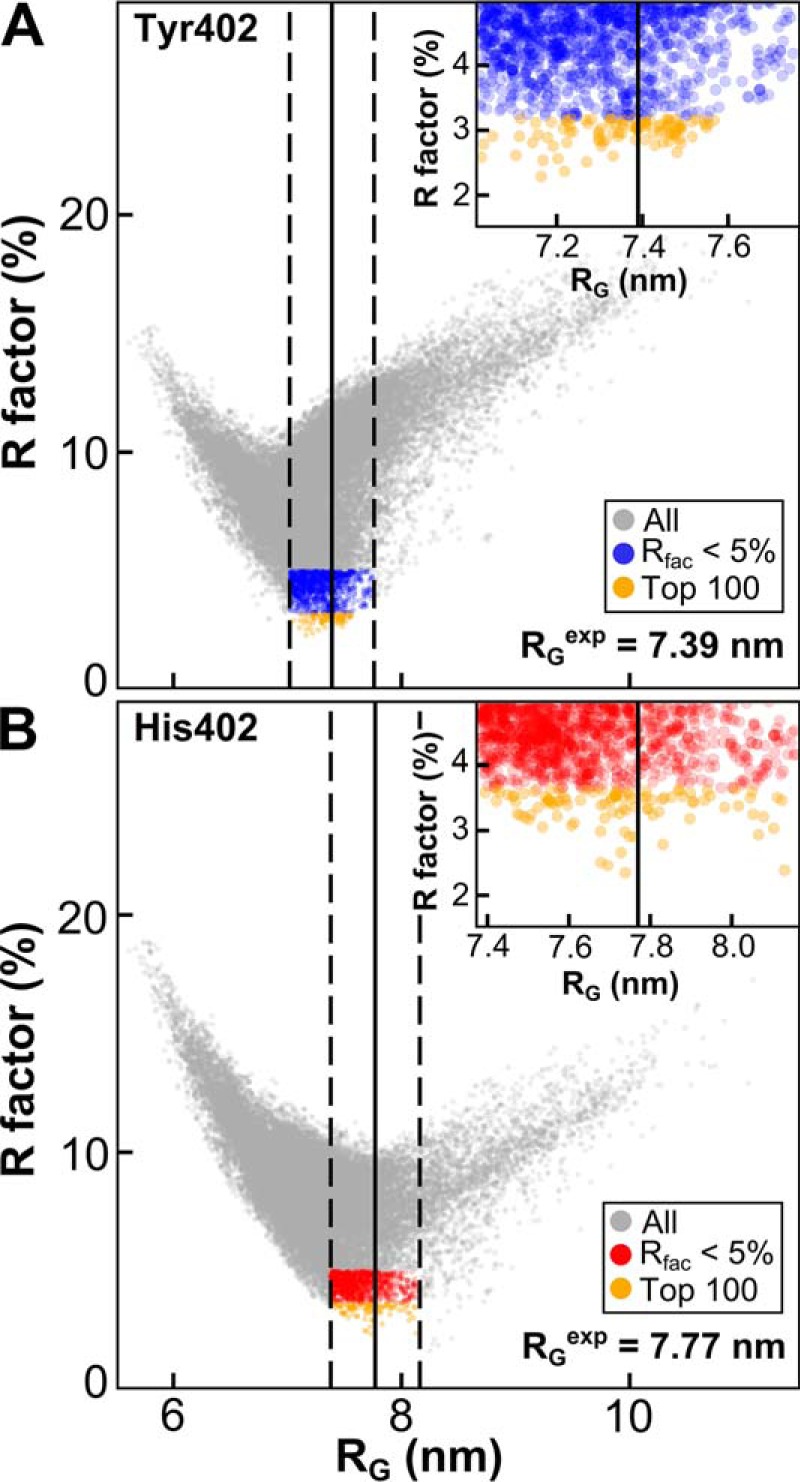
**Atomistic modeling searches for the FH solution structure.** The 29,715 FH models were fitted to one experimental scattering curve for each of homozygous FH Tyr-402/Val-62 (*A*) and FH His-402/Val-62 (*B*), each extrapolated to zero concentration. *Gray* corresponds to all FH models. The *blue/red* subsets corresponds to FH models that passed two filters, namely *R_g_* within ±5% of the experimental *R_g_* and an *R*-factor of <5%. *Orange* corresponds to the 100 best-fit FH models (see *inset*). The *vertical black lines* represent the experimental *R_g_* for FH Tyr-402 (7.39 nm) and FH His-402 (7.77 nm). The *dashed lines* represent the ±5% upper and lower boundaries of these *R_g_* values.

The atomistic modeling of the two FH Tyr-402 and two His-402 scattering curves was initiated using 17 high-resolution SCR structures, three SCR homology models, and eight glycan chains to construct an energy-minimized initial FH model (Fig. 1 and Tables 1 and 2; see under “Materials and methods”). By varying the torsion angles at eight inter-SCR linkers, 510,000 FH models were created in four Monte Carlo simulations, from which 29,715 models were retained for analysis as being physically realistic without steric clashes. Comparison of the four extrapolated experimental scattering curves with the 29,715 theoretical curves gave an *R*-factor *versus R_g_* distribution with a minimum close to the experimental extrapolated *R_g_* value (Fig. 8). An *R_g_* filter of ±5% of the experimental value and an *R*-factor filter of >5% gave 1240/2678 and 1219/310 best-fit models for the two Tyr-402 and two His-402 allotypes, respectively; these are 1–9% of the 29,715 trial models (*blue/red,* Fig. 8). The best-fit 100 FH models were selected on the basis of the lowest *R*-factors (*orange,* Fig. 8) of 2.3–3.2 and 2.3–3.6%, respectively (Table 3).

**Table 2:** **Homology modeling of the SCR-9, SCR-14, and SCR-17 domains**

Target domain	Template domain	PDB code	Residues	Maximum score	Total	Query cover	E value	Residue identities
						%		%
SCR-9	SCR-4	2UWN	148–186	40.1	41.2	68	0.0004	44
SCR-14	SCR-15	3ZD1	5–59	37.5	37.5	100	0.002	36
SCR-17	SCR-18	3SW0	3–59	42.2	77.6	100	0.00007	37

**Table 3:** **Experimental X-ray and analytical ultracentrifugation data for FH Tyr-402/Val-62 and His-402/Val-62 and their modeling fits**

	Filter	Models	*R_g_*	*R_XS_*_-1_	*R_XS_*_-2_	*L*	*R* factor	*s*_20, *w*_^0^
			*nm*	*nm*	*nm*	*nm*	%	*S*
**Experimental data**								
FH Tyr-402/Val-62			7.39 ± 0.25	2.21 ± 0.06	1.77 ± 0.01	28–29		5.66 ± 0.08
			7.35 ± 0.13	2.27 ± 0.06	1.77 ± 0.01			
			7.61 ± 0.01*^a^*					
FH His-402/Val-62			7.77 ± 0.27	2.02 ± 0.06	1.76 ± 0.01	26–28		5.69 ± 0.02
			7.22 ± 0.15	2.15 ± 0.06	1.75 ± 0.01			
			7.54 ± 0.01*^a^*					

**Atomistic modeling**								
Library of 29,715 FH models	None	29,715	5.61–11.08	NA*^b^*	NA	NA	2.8–25.3	NA
FH Tyr-402/Val-62 (sample 1)	*R_g_* and *R-*factor	1240	7.02–7.75	NA	NA	NA	2.3–4.9	NA
	Best fit	100	7.02–7.57	2.34–2.98	1.85–2.10	NA	2.3–3.2	5.35–5.77
	PCA group 2 (NT out)	49	7.34–7.57	2.34–2.62	1.95–2.10	NA	2.7–3.2	5.38–5.77
	Centroid	1	7.41	2.50	2.03	NA	3.1	5.52
FH Tyr-402/Val-62*^c^* (sample 2; data not shown)	*R_g_* and *R-*factor	2,678	6.98–7.72	NA	NA	NA	1.9–4.9	NA
	Best fit	100	7.03–7.47	2.71–3.05	1.81–2.04	NA	1.9–2.7	5.33–5.70
	PCA group 14 (NT in)	48	7.11–7.43	2.71–2.98	1.81–1.99	NA	1.9–2.7	5.36–5.70
	Centroid	1	7.32	2.84	1.95	NA	2.2	5.49
FH His-402/Val-62 (sample 1)	*R_g_* and *R*-factor	1,219	7.38–8.16	NA	NA	NA	2.3–4.9	NA
	Best fit	100	7.39–8.13	2.32–3.15	1.48–1.97	NA	2.4–3.6	5.21–5.61
	PCA group 5 (NT in)	79	7.39–7.95	2.67–3.07	1.64–1.94	NA	2.5–3.6	5.21–5.55
	Centroid	1	7.48	2.92	1.87	NA	3.2	5.44
FH His-402/Val-62 (sample 2; data not shown)	*R_g_* and *R*-factor	310	6.86–7.58	NA	NA	NA	2.7–4.9	NA
	Best fit	100	6.86–7.57	2.09–2.93	1.82–2.10	NA	2.7–4.0	5.38–5.88
	PCA group 19 (NT out)	84	6.86–7.57	2.28–2.82	1.89–2.10	NA	2.7–4.0	5.38–5.78
	Centroid	1	7.40	2.48	2.03	NA	3.8	5.51

*^a^* The *R_g_*, *R_xs_*, and *L* values correspond to the SAXS curve extrapolated to zero concentration. The two *R_g_* values correspond to the Guinier fits; the third corresponds to the *P*(*r*) analyses.

*^b^* NA is not available.

*^c^* In this instance I62V was heterozygous; the other three were homozygous for Val-62.

The *s*_20, *w*_^0^ values of the best-fit 100 FH models were calculated using HYDROPRO to be 5.35–5.77 and 5.21–5.61 S and for the centroid models to be 5.49–5.52 and 5.44–5.51 S, both for FH Tyr-402 and FH His-402, respectively (Table 3). These values agreed well with the experimental values of 5.66–5.69 S (Table 3) and confirmed the outcome of the atomistic modeling, given that the mean difference between the modeled and experimental values should be ± 0.21 S for related macromolecules, including antibodies (63). Like antibodies that are also considered to be flexible molecules, HYDROPRO was effective in calculating the *s*_20, *w*_^0^ values; this was attributed to the use of the averaged best-fit solution structures. The calculations were much improved to those of the 2009 FH modeling, which gave 4.91–5.01 S from older scattering models that in retrospect were too elongated (26).

### NT-COM and CT-COM separation distributions

**Figure 9.**
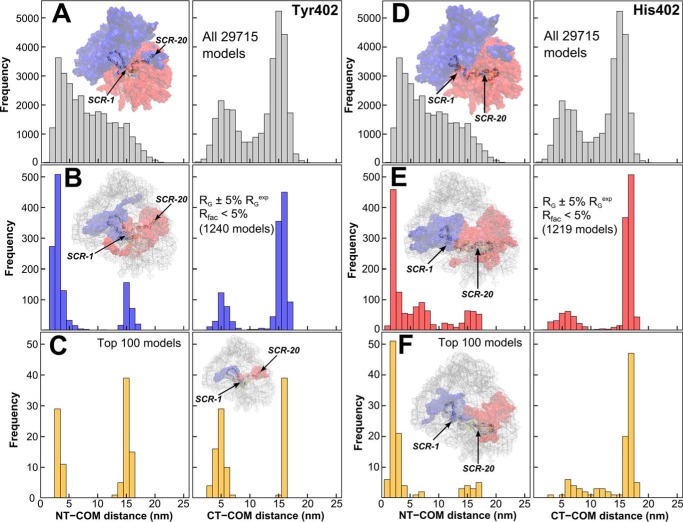
**Center-of-mass separation frequencies in the FH Tyr-402 and FH His-402 models.** *A* and *D*, *top two panels* (*gray*) show the separations (NT-COM and CT-COM) between the N-terminal α-carbon (*NT*) and C-terminal α-carbon (*CT*) to the center of mass (COM) for all 29,715 FH models. *B* and *E,* separations are shown for the filtered 1240 and 1219 FH models (Fig. 8). *C* and *F,* separations are shown for the 100 best-fit FH models. For each set of FH models, the density plot shows the N-terminal half in *blue*, the C-terminal half in *red*, and the best-fit model as a *cartoon.*

The best-fit SCR arrangements within the 29,715 trial FH models were shown to be bimodal using histograms of their N-terminal α-carbon (NT) and C-terminal α-carbon (CT) to COM separations (Fig. 9). Longer separations corresponded to extended SCR arrangements in FH; shorter separations corresponded to a folded-back bent one.

(i) For all 29,715 models, the NT-COM and CT-COM separations corresponded to similar ranges of 0.5–21.8 and 0.8–22.4 nm, respectively (Fig. 9, *A* and *D*). However, the most populated NT-COM separations were short ones (3–5 nm) that tailed off after >16 nm. In distinction, the most populated CT-COM separations were long ones (14–16 nm) followed by short ones (∼5 nm) (Fig. 9, *A* and *D*). The NT-COM separations were right-skewed with a skewness coefficient of 0.53, whereas the CT-COM separations were left-skewed and bimodal with a skewness coefficient of −0.55. Overall, the separation distributions were uneven and not normally distributed. This unexpected outcome was attributed to the seven glycans in SCR-12/18 that perturbed the random generation of physically-realistic FH structures (Fig. 1) and the asymmetric distribution of the longest inter-SCR linkers in FH (26).

(ii) For the 1219–1240 filtered FH models, both the NT-COM and CT-COM distance distributions corresponded to small subsets of those for all 29,715 models (Fig. 9, *B* and *E*). Density plots showed that these models occupied limited conformations when compared with all 29,715 models (*insets,* Fig. 9, *B* and *E*). The separations for both allotypes were bimodal, being left-skewed for the NT-COM distances and right-skewed for the CT-COM distances. Overall, no differences were seen between the two allotypes.

(iii) For the two sets of 100 best-fit FH models, the two NT-COM and CT-COM distance distributions were again largely bimodal distributions (Fig. 9, *C* and *F*). Similar bimodal results were obtained for the fits with the other pair of FH Tyr-402 and FH His-402 experimental curves (distributions not shown).

For the filtered and top 100 models, statistical analyses were performed to confirm that the separation distance distributions (Fig. 9) were unaffected by sampling bias. The best-fit 100 models were not necessarily a converged subset of the filtered models as seen in other modeling (64). A nonparametric (*i.e.* no underlying assumption) Kolmogorov-Smirnov test (data not shown) was thus used to indicate that the best-fit distances for NT-COM and CT-COM were either short or long and were independent of any sampling bias observed in all 29,715 models. This suggested that FH existed in two conformations.

**Figure 10.**
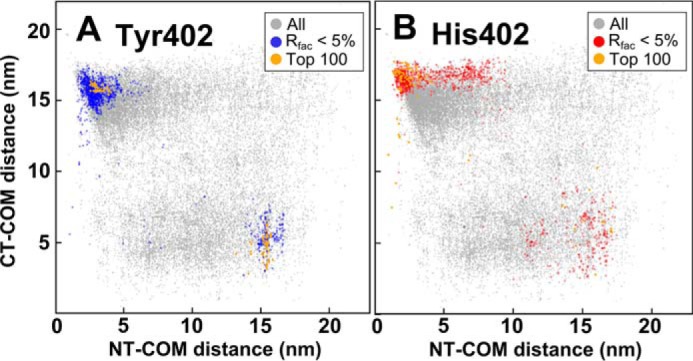
**Separation densities in the FH Tyr-402 and FH His-402 models.** For each of the Tyr-402 (*A* and *B*), joint NT-COM and CT-COM separations for the FH Tyr-402 and FH His-402 models are compared with each other. *Blue/red* corresponds to the filtered FH models, and *orange* corresponds to the 100 best-fit FH models (Fig. 8).

The full FH SCR domain arrangement was examined using pairs of NT-COM and CT-COM distances. The 29,715 separations were nonrandomly distributed between 1 and 25 nm, showing that a broad range of FH conformations had been generated as desired (Fig. 10, *A* and *B*). The most sampled best-fit NT-COM and CT-COM separations occurred at 1–10 and 12–17 nm, respectively, at the top left-hand corner. The second most sampled best-fit separations occurred as a broad cluster at 5–17 and 2–8 nm, respectively. For the filtered Tyr-402 models, two structural regions were visible as two dense clusters in *blue*, in which the top 100 Tyr-402 models were shown in *orange* (Fig. 10*A*). For the filtered His-402 models, the same two clusters were seen in *red* and *orange*, respectively (Fig. 10*B*). In contrast, FH models with long NT-COM and CT-COM separations (>15 nm) showed large radii of gyration and poor fits with *R*-factors >5% (*gray,* Fig. 10, *A* and *B*). This analysis showed that FH either has a bent-inwards N-terminal region and an extended C-terminal region or vice versa.

### Best-fit conformations for FH Tyr-402 and His-402

**Figure 11.**
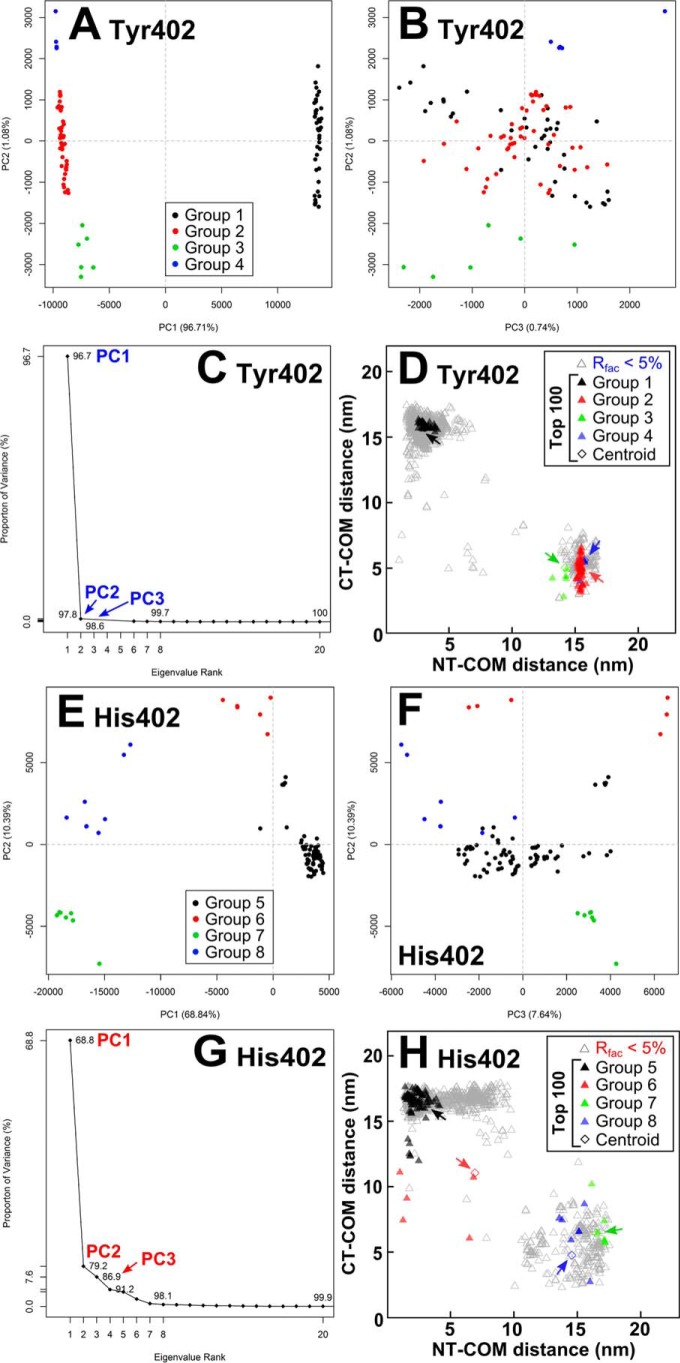
**Principal component analyses of the 100 best-fit models for FH Tyr-402 and FH His-402.** For FH Tyr-402 (*A* and *B*) and FH His-402 (*E* and *F*), the 100 best-fit models were grouped by PCA into four groups, 1–4 (*black, red, green,* and *blue*, respectively), as exemplified by the first three principal components (PC2 *versus* PC1 and PC3 *versus* PC2). *C* and *G,* first three eigenvalue rankings (PC1 to PC3) accounted for variances of 98.5 and 86.9% in the 100 best-fit FH models. *D* and *H, black, red, green,* and *blue triangles* correspond to the PCA groups 1–4 and 5–8 in each set of best-fit 100 models. The *four arrowed diamonds* correspond to the average (centroid) model for each PCA group.

After superimposition of the top 100 Tyr-402 and His-402 FH structures, visual inspection showed multiple conformers. To understand these conformers, they were clustered into conformational families using principal component analysis (PCA) (Fig. 11) (65). PCA determines the correlated motions of protein residues as linearly uncorrelated variables termed principal components (66). These “essential motions” are extracted from a covariance matrix of the atomic coordinates of the frames in the trajectory. The eigenvectors of this matrix each have an associated eigenvalue that characterizes a mode of the motion or variance.

**Figure 12.**
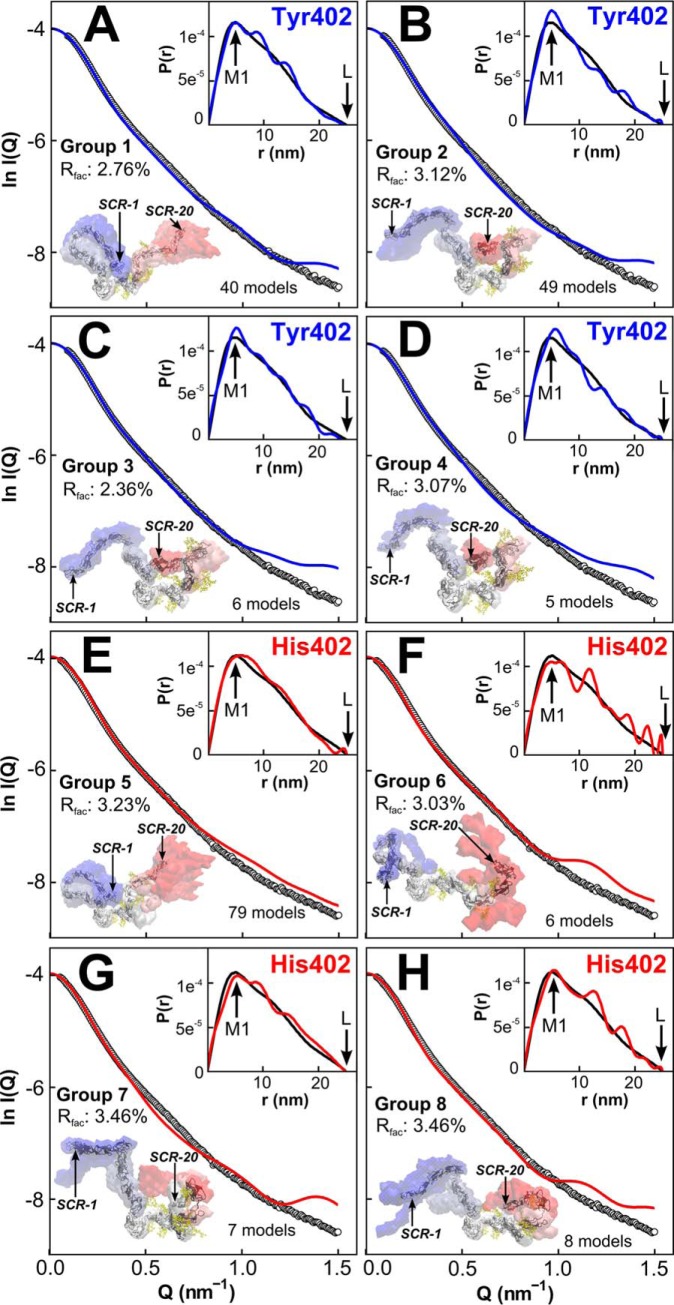
**Scattering curve fits for the centroid PCA models for FH.** For FH Tyr-402 (*A–D*) and FH His-402 (*E–H*). the four panels display the centroid FH model for the PCA groups 1–4 and 5–8, respectively, in density plots. The number of FH models in each PCA group is displayed. The experimental curve is denoted by *black circles*, and the theoretical curve is denoted as *solid blue/red lines*. The *inset* shows the *P*(*r*) curves for the experimental data (*black line*) and modeled curve (*blue/red line*). For each PCA group, the conformational space is represented as *blue* (NT), *white* or *red* (CT) density. The average (centroid) model of each PCA group is depicted as a *black cartoon* in which SCR-1 and SCR-20 are *arrowed*.

The PCA analyses confirmed that two families of FH structures with either a bent-inwards N-terminal region and an extended C-terminal region, or vice versa, were seen. Four PCA groups accounted for 98.5 and 86.9% of the variances in the top 100 models of each Tyr-402 and His-402, respectively (Fig. 11, *C* and *G*). For each PCA group, the combined NT-COM and CT-COM separation distances were plotted (Fig. 11, *D* and *H*). The conformational density of each group was represented as either *blue* (NT) or *red* (CT) density, with the centroid FH model depicted as a *black cartoon* (Fig. 12). The 60 Tyr-402 models in PCA groups 2–4 corresponded to an extended N terminus and a bent inwards C terminus (*red, green,* and *blue,* Figs. 11*D* and 12, *B–D*). The 40 Tyr-402 models in PCA group 1 corresponded to a bent inwards N terminus and an extended C terminus (*black,* Figs. 11*D* and 12*A*). The 79 His-402 models in PCA group 5 corresponded to a bent inwards N terminus and an extended C terminus (*black*, Figs. 11*H* and 12*E*). The 21 His-402 models in PCA groups 6–8 corresponded to a bent inwards C terminus and an extended N terminus (*red, green,* and *blue*, Figs. 11*H* and 12, *F–H*).

Each of the PCA groups 1–8 gave FH structures whose theoretical scattering curves *I*(*Q*) fitted well with the experimental Tyr-402 or His-402 curves with low *R*-factors between 2.4 and 3.8% (Fig. 12). The visual agreement was good to at least *Q* = 1.0 nm^−1^. The centroid models with an extended N terminus and a bent inwards C terminus (Fig. 12, *B–D,* and *F*) gave fits similar to those with a bent inwards N terminus and an extended C terminus (Fig. 12, *A, E, G,* and *H*). The theoretical distance distribution curves *P*(*r*) also showed good agreements with the experiment. Thus, the eight centroid FH models gave very similar *M1* peak values that agreed with the experimental *P*(*r*) curves, where *M1* was 4.7–5.0 nm (Fig. 7*C*). Their intensities were comparable, although these showed oscillations around the experimental *P*(*r*) curves. Of note, the experimental *P*(*r*) curves generally appeared smoother than the theoretical curves. This is likely due to both the lower resolution and the fluctuation between the alternative conformations of the solution structure studied by the experimental curves. Only eight of the 200 best-fit structures had conformations in which both the N- and C-terminal regions were inwardly bent, and none showed that both regions were extended in any structure. The same PCA analyses for the other pair of available Tyr-402 and His-402 curves gave similar structural outcomes (data not shown).

**Figure 13.**
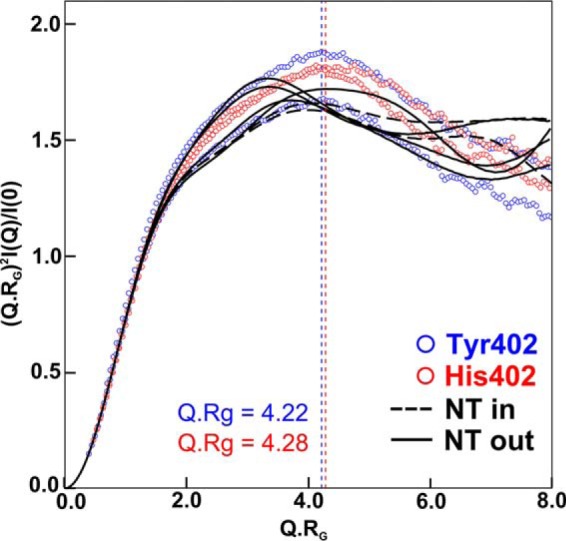
**Normalized Kratky plots for the experimental and best-fit FH curves.** The four experimental curves are shown in *blue* and *red* for the Tyr-402 and His-402 allotypes, respectively, which peak at *Q*·*R_g_* values of 4.22 and 4.28, respectively. For both allotypes, the theoretical curves for four N-terminal extended and two C-terminal extended FH models are shown as *black solid* and *dashed lines*, respectively. These correspond to the two sets of centroid models of Fig. 12, *B, C, D,* and *H* and Fig. 12, *A and E, respectively*.

The normalized Kratky analyses for six modeled best-fit curves corresponding to the NT-in and NT-out centroid conformations from Fig. 12 each showed a clear peak that was similar in shape to the experimental curves (Fig. 13). The *Q*·*R_g_* range of the modeled peaks were 3.90–4.12 and 3.33–4.30, respectively, in good accord with the observed values of 4.22–4.48. This similarity showed that the modeling had mostly replicated the extended flexible multidomain protein structures. The reduced *Q*·*R_g_* peak positions for some of the models may indicate an insufficient representation of flexible extended linkers in FH. Overall, this comparison validated our analyses that FH is a multidomain protein whose domains were connected by semi-extended linkers (62).

## Discussion

FH is an essential regulatory glycoprotein that protects host cells from complement activation both at the cell surface and in the fluid phase. In this study, the most accurate molecular models to date have been produced for full-length FH, and these suggest that FH exists in two distinct conformations that enable its complement functions. Common and rare genetic variants in FH are associated with major and rare inflammatory diseases, including AMD, Alzheimer's, aHUS, and C3G (34). For AMD, the Y402H and V62I polymorphisms are associated with increased and decreased risk, respectively. To perform its regulatory activities, FH binds to multiple physiological ligands, including to itself, with weak *K_D_* values in the micromolar range, and has a highly glycosylated 20-domain structure (Fig. 1). For FH, characterization of its self-association is important for determining its solution conformation and investigating the development of drusen in AMD. Up to now, the self-association of FH has only been characterized for native heterozygous FH purified from plasma, and this has not been widely accepted. Likewise, the only FH molecular structures up to now had been determined by preliminary modeling of X-ray scattering curves, resulting in two different folded-back structures whose significance was unclear (26, 51, 67). Here, working with genotyped FH, we have now shown using four independent methods that WT FH Tyr-402 and risk-associated FH His-402 both significantly self-associate. The V62I polymorphism involves a minor structural rearrangement within the core of SCR-1 and is protective for AMD due to its subtly better capacity to bind C3b, inhibit proconvertase formation, and catalyze inactivation of fluid-phase and surface-bound C3b (45, 56). For both polymorphisms, it was important to demonstrate that neither affected FH oligomerization by self-association. More importantly, by working with genotyped FH samples with reduced sample aggregation, and also by utilizing a much-improved atomistic modeling procedure based on molecular dynamics and Monte Carlo procedures and statistical assessments of the outcome, we have determined similar detailed molecular models for both allotypes of full-length FH, including its eight FH glycans. Our statistical validation of two alternative FH conformations significantly improves our understanding of ligand binding to FH and how this may be perturbed in its function.

### Self-association of FH

FH self-association is important for FH function because this will increase the local concentration of its ligand-binding sites. FH oligomer formation in solution was first observed by X-ray scattering (28). Another FH study at that time used EM *in vacuo* and gel filtration to deduce that FH was monomeric; however, the low concentrations in use in that study would not have favored dimer formation, and gel filtration is insensitive to FH self-association (Fig. 2) (33). The FH fragments SCR-6/8 and SCR-16/20 each formed dimers (26, 29–31). This outcome predicted that full-length FH would form weak daisy-chained multimers through these two independent dimerization sites. These multimers were indeed observed for heterozygous FH previously by AUC as a series of small peaks at large S values, in which the sedimentation equilibrium fits suggest that FH dimers and trimers are initially formed reversibly below 1 mg/ml (26, 29). Higher FH concentrations favor the formation of larger oligomers by further daisy-chaining to result in irreversible oligomers that can be removed by size-exclusion chromatography. Here, these small peaks were now observed again for homozygous FH at 1.1 mg/ml (Fig. 3*C*). Biologically, given that the plasma concentration of FH is around 0.7 mg/ml (19), reversible dimers and trimers are expected to form *in vivo*. The resulting clustering of FH on cell surfaces, when bound to surface markers and C3b/C3d, is likely to enable broader surface protection, thus being functionally significant (68). However, the accumulation of FH by clustering through self-association may also relate to the formation of soft drusen in AMD via chronic inflammation, in particular if FH oligomer formation becomes irreversible (69). Consistent with this, studies with full-length FH showed that its affinity for both heparin and HS was not altered by the Y402H polymorphism (39, 70, 71). It was also found that FH multimer formation is enhanced in the presence of zinc, which inhibits FH function (57). In this context, the self-association of FH Tyr-402 and FH His-402 becomes of interest. In this study, AUC showed that 11.7–12.8% multimers were present for FH His-402 and FH Tyr-402 (Fig. 3*D*).

Because the occurrence of FH self-association had been questioned (33, 72), other orthogonal techniques were utilized in our study to confirm this. MS confirmed the existence of as much as 23–28% FH dimer and trimer for both FH allotypes (Fig. 4). SPR revealed self-association for both full-length FH allotypes and for both SCR-6/8 allotypes (Fig. 5). SAXS analyses of the Guinier *I*(0)/*c*, *R_g_*, and *R_XS_*_-1_ parameters showed clear concentration effects attributable to self-association (Fig. 7, *D–F*). One immediate consequence of this outcome was the need to allow for self-association effects in the SAXS structural studies to model the monomeric FH structure. It was necessary to extrapolate the scattering curves to zero concentration. FH self-association has had a marked effect on earlier SAXS data collection. The first FH studies gave an *R_g_* value of 12.4 nm in 1991 (28), which was reduced to 11.4 nm in 2001 (50), then to an extrapolated value of 8.90 nm in 2008 (26, 29), and now to an extrapolated value of 7.22–7.77 nm (Fig. 7*E*). This reduction in *R_g_* is attributed to reduced aggregation in FH because of improved protein handling (*e.g.* FH forms aggregates in storage or when in transit); its occurrence may have caused the lack of progress with the protein crystallization of FH to date.

### Atomistic modeling of two FH conformations

**Figure 14.**
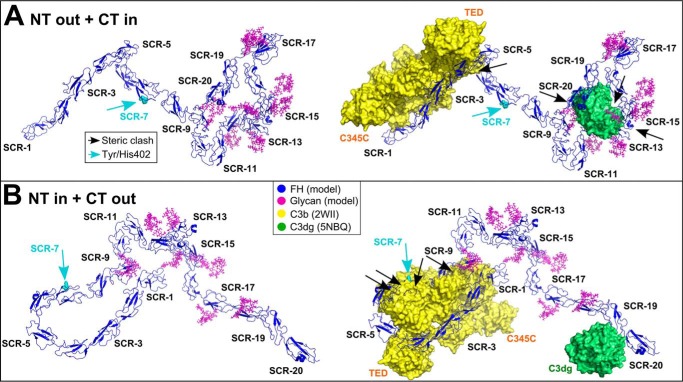
**Best-fit FH centroid models superimposed onto C3b and C3dg.** For both allotypes, the most representative N-terminal extended (*A*) and C-terminal extended (*B*) FH models are shown as *blue ribbon traces* (*left*). These correspond to the centroid models shown in Figs. 12, *B* and *E*, respectively. The coordinates are available for download in supporting Materials. These models were superimposed onto the SCR-1/4-C3b and SCR-19/20-C3dg crystal structures, in which C3b is shown as a *yellow* surface and C3dg is shown as a *green surface* (*right*, PDB codes 2WII and 5NBQ). The eight FH glycans are shown in *magenta*. The *cyan arrow* indicates Tyr-402/His-402. *Black arrows* indicate steric clashes of FH with C3b or C3dg.

The new molecular modeling of the FH scattering curves has benefitted from five improvements (54) as follows: (i) the application of molecular dynamics to generate an initial full FH structure; (ii) the use of 17 high-resolution SCR structures, in distinction to up to 11–14 SCR structures previously; (iii) the inclusion of eight energy-refined glycan chains in FH; (iv) the use of rapid Monte Carlo simulations with the inter-SCR linkers to generate a large number of 29,715 physically realistic trial FH structures; and (v) statistical analyses of the resulting ensemble of best-fit structures. Interestingly, our previous two 2008 modeling studies had yielded alternative folded-back FH structures with either the N- or C-terminal SCR domains in an extended conformation (26, 51, 67), yet their significance was unclear. Here, the statistical analysis of the 29,715 curve fits showed that both alternative FH conformations were valid outcomes from the SAXS modeling (Fig. 14, *A* and *B*). In the 200 best-fit structures, some 81 and 119 structures, respectively, corresponded to (i) an extended N terminus and a bent inwards C terminus, and (ii) a bent inwards N terminus and an extended C terminus. In principal, either of these two FH structures may be the actual solution structure, or both FH structures may co-exist as an equilibrium in solution. Consideration of C3b and C3d binding (below) suggested both FH conformations exist. Further statistical analyses of the 200 best-fit structures showed that the two FH Tyr-402 and His-402 allotypes displayed the same solution structures.

### Biological significance of the FH Tyr-402 and His-402 models

To summarize FH activity, SCR-1/5 is involved in both cofactor and decay-acceleration activities (73, 74), whereas SCR-19/20 is involved in the recognition of host cell surfaces (75). These two functions are mediated by N-terminal SCR-1/4 binding to both C3b and FI and C-terminal SCR-19/20 binding to C3d, heparin, and sialic acid. C3dg and C3d, being two fragments of C3 that each contain the thioester domain for host-surface binding, are formed by the breakdown of C3b by FI and/or serum proteases, and both bind to complement receptor 2 to trigger immune complex clearance and to stimulate the immune response (1). C3d is also an autologous helper T-cell target (76). During complement regulation at host cell surfaces, an increase in the number of C3d-binding sites for SCR-19/20 would enable more FH to be recruited at the host cell surface for a more rapid breakdown of C3b (77). For cell-surface attachment of FH, an optimal combination of affinities for each of C3b and polyanion ligand is essential for C-terminal FH binding (68), for which both SCR-7 and SCR-19/20 bind bivalently via heparin (21, 27).

The functions of FH were clarified by superimposing the two alternative best-fit conformations of FH with crystal structures for the SCR-1–4-C3b complex and the SCR-19–20-C3dg complex (PDB codes 2WII and 5NBQ) (52, 78).

(i) The N-terminal extended FH conformation accounted for binding to C3b at SCR-1/4, and to heparin and sialic acid, but less so for C3dg. The binding of C3b to SCR-1/4 showed minimal steric clashes with the rest of FH (*black arrow,* Fig. 14*A*). Likewise, for binding to fluid phase FI, the SCR-1/3 domains were accessible, including the FH residues 101, 106, 112, and 119 that were important for this interaction (52). For bivalent SCR-7 and SCR-19/20 binding to heparin and/or sialic acid on cell surfaces, the essential residues in each of SCR-7 (Tyr/His-402) and SCR-19/20 (FH residues 1181–1183, 1191, 1195, 1196, 1198, and 1199) were solvent-accessible. However, C3dg binding to SCR-19 resulted in significant steric clashes with SCR-9, SCR-12/15, and the glycans (*black arrows,* Fig. 14*A*), implying that some structural rearrangement will be required for C3dg binding.

(ii) In contrast, the C-terminal extended FH model accounted for binding to C3dg at SCR-19 or SCR-20 and to heparin and/or sialic acid on cell surfaces but not to C3b at SCR-1/4. The binding of C3b to SCR-1/4 resulted in large steric clashes with the SCR-6/8 domains (*black arrows,* Fig. 14*B*). The shortest distance between the SCR-1/4 and SCR-6/9 domains was ∼6 nm between SCR-1 and SCR-9. For binding to fluid phase FI, the SCR-1/3 domains were accessible. The accessibility of the Tyr/His-402 residue in SCR-7 was not compromised. For bivalent binding to heparin and/or sialic acid on cell surfaces, each of SCR-7 and the essential residues in SCR-19/20 (above) were accessible. For C3dg binding to SCR-19 or SCR-20 (79), no steric clash was seen (Fig. 14*B*).

In summary, the two extended N-terminal and extended C-terminal FH models (Fig. 14, *A* and *B*) showed that SCR-7 and SCR-19/20 were spatially aligned to permit simultaneous bivalent FH binding to glycosaminoglycans at the host cell surface. However, in order for FH to bind C3b via SCR-1/4, potential steric hindrance from SCR-6/8 would need to be removed, most likely by moving the flexible linkers between SCR-4/5 and SCR-5/6 (52). Such large conformational changes in other complement proteins have already been reported for the thioester domain in C3b, and the Bb fragment from C3b within the C3 convertase (52). Interestingly, in the FH model with the compact N terminus, the smallest distance between SCR-1/4 and SCR-6/9 involved Arg-78 in SCR-1 and Asp-538 in SCR-9, which bear opposite charges (80). We speculate that these domains may electrostatically attract each other; indeed, conformational changes in FH with increase in ionic strength have been reported (26). Further studies are required to investigate such a proposed mechanism.

The existence of two FH conformations with different C3b and C3d binding strengths accounts for previously reported *K_D_* values for each of SCR-1/4 binding to C3b (10–14 μm) and SCR-19/20 binding to C3b or C3d (1–5 and 0.2–8 μm, respectively). These *K_D_* values are similar to the *K_D_* value of full-length FH binding to C3b (0.6–3 μm) (21), indicating no synergy between the two binding sites at SCR-1/4 and SCR-19/20, and meaning that these two C3b and C3d sites are independent of each other. This outcome is in concord with our N-terminally extended and C-terminally extended FH models that bind to C3b or C3d separately but not both together. In summary, FH is seen with an N-terminally extended conformation that is able to dismantle fluid phase C3 convertases, and also with a distinct C-terminally extended conformation that binds to cell surfaces via deposited C3d for enhanced local protection from complement activation. Both alternative conformations are able to bind to glycosaminoglycans at the host cell surface, thus protecting this against excess C3b activity. Overall, the SAXS data showed that FH underwent significant self-association, and the modeling of these data for the monomer has resulted in the proposal of two distinct FH conformations that would enable FH to perform separate C3b or C3d binding functions in either the fluid phase or at host cell surfaces, respectively.

## Materials and methods

### Protein purification of FH and FH SCR-6/8

Thirty aliquots of 50–100 ml of human blood was collected from anonymous healthy volunteers with ethical approval (University College London, ID: 6986/001). The studies abide by the Declaration of Helsinki principles. The Y402H and I62V polymorphisms were identified by direct DNA sequencing of their PCR product. PCR was performed with Extensor Hi-Fidelity PCR® master mix from ABgene following the manufacturer's protocol. Bidirectional sequencing of the PCR product was carried out with Big Dye Terminator version 3.1 on a 3730 DNA Analyzer (Applied Biosystems, Cheshire, UK). Homozygous FH Tyr-402, which was homozygous or heterozygous at I62V, and double homozygous FH His-402/Val-62 were purified using monoclonal affinity chromatography with an MRC-OX23–Sepharose column (29, 81). Bound FH was eluted from the column using 3 m MgCl_2_, pH 6.9, and then dialyzed into HEPES buffer (10 mm HEPES, 137 mm NaCl, pH 7.4) in the presence of 0.5 mm EDTA to remove Mg^2+^. To avoid cross-contamination between different FH samples, the MRC-OX23 column was washed with guanidine (0.2 m Tris, 4 m guanidine-HCl, pH 8.0) between purifications. Each FH sample was passed through a Hitrap^TM^ protein G HP column to remove residual IgG contaminant. Nonspecific aggregates and human serum albumin were removed by gel filtration on a Superose^TM^ 6 prep grade XK 16/60 column (Fig. 2). Protein concentrations were determined using an absorption coefficient of 16.2 for FH (Tyr-402) and 16.1 for FH (His-402) (1%, 280 nm, 1 cm path length), revised in the light of its known glycosylation (26). After sedimentation velocity experiments on the purified FH allotypes, the proteins were re-passed through the Superose^TM^ 6 prep grade XK 16/60 column to analyze the presence of FH oligomers (Fig. 2).

Recombinant FH SCR-6/8 was purified as described previously (30). SCR-6/8 Tyr-402 was expressed in BL21 (DE3) *Escherichia coli* cells using a pET21ab vector system, whereas SCR-6/8 His-402 was expressed in BL21 (DE3)pLysS *E. coli* cells using a pET14b vector system. The protein was solubilized from inclusion bodies, and the refolded protein was passed through a Hitrap^TM^ 5-ml heparin HP column equilibrated in Tris buffer (50 mm Tris, 150 mm NaCl, 1 mm EDTA, pH 7.4). The bound protein was washed with Tris buffer and eluted with up to 1 m NaCl in Tris buffer. Proteins were dialyzed into HEPES buffer for experiments, and their integrity was routinely checked by SDS-PAGE before and after scattering and ultracentrifugation.

### Sedimentation velocity data

AUC data were obtained on two Beckman XL-I instruments equipped with AnTi50 and AnTi60 rotors. Sedimentation velocity experiments were performed at 20 °C at rotor speeds of 50,000 and 60,000 rpm in two-sector cells with column heights of 12 mm for five FH Tyr-402 samples at 0.5, 1.1, and 2.1 mg/ml (3.2–13.6 μm), and repeated for five FH His-402/Val-62 samples, all in HEPES buffer. Size-distribution analyses *c*(*s*) were performed using SEDFIT (82, 83). SEDFIT provided shape and size data using direct boundary Lamm fits of up to 300 scans using a fixed resolution of 200, and floated the meniscus, the bottom of the cell, the baseline, and the average frictional ratio *f/f*_0_ (initial value 1.781), until the overall root mean square deviation and agreement between the observed and calculated sedimentation boundaries were satisfactory. The percentage fraction of oligomers in the total loading concentration was derived using the *c*(*s*) integration function. Other details are described elsewhere (26, 29).

### Mass spectrometry data

Mass spectra were acquired on a Q-ToF (quadrupole-TOF) 2 MS instrument for high mass operation and equipped with a Z-spray nanoflow source (Waters, Manchester, UK) (84). The following instrumental parameters were used: capillary voltage 1.7 kV; cone voltage 80–120 V; cone gas 100 liters/h; collision cell voltage up to 200 V; ion transfer stage pressure 0.004–0.02 mbar; and argon collision gas at a collision cell pressure of 2.5 bar. Mass spectra were analyzed using the software Data-Explorer. Purified FH samples were concentrated to a final concentration of about 4 mg/ml (26 μm), and then dialyzed overnight into 140 mm or 1 m ammonium acetate at pH 7.5 for measurement the next day. One pair of FH Tyr-402 and His-402 allotypes was analyzed in both buffers.

### Surface plasmon resonance data

The binding of the FH Tyr-402 and His-402 allotypes to immobilized FH and that of the SCR-6/8 Tyr-402 and His-402 allotypes to immobilized FH SCR-6/8 were studied using a Biacore X100 with version 1.1 of its evaluation software (GE Healthcare, Uppsala, Sweden). For both the self-association studies and the human serum albumin control, FH was coupled to a carboxymethylated dextran (CM3) research grade sensor chip with a shorter carboxymethylated dextran matrix than a standard CM5 chip, which gave nonspecific binding (85). Each FH SCR-6/8 allotype was coupled to a CM4 research grade sensor chip with a low degree of carboxymethylation compared with a CM5 chip. Immobilizations utilized a standard amine coupling procedure according to the manufacturer's protocol. FH (20 μg/ml) or SCR-6/8 (10 μg/ml) in 10 mm acetate buffer, pH 5.5 (FH), was injected over flow cell 2 to reach a maximum response of 150 RU. A control surface cell was prepared identically on flow cell 1 but without protein immobilization. Equilibrium analyses were performed at 25 °C using Biacore X100 wizards at a flow rate of 30 μl/min. The running buffer was HEPES buffer. FH Tyr-402 was injected for 180 s at six concentrations between 0.1 and 1.5 mg/ml (0.7–9.7 μm). FH His-402 was injected at six concentrations between 0.1 and 1.8 mg/ml (0.7–11.7 μm). SCR-6/8 Tyr-402 was injected for 150 s over the CM4 chip immobilized with either the Tyr-402 or the His-402 allotype at concentrations of 0.1–0.8 mg/ml (5–40 μm). SCR-6/8 His-402 was injected over the Tyr-402 CM4 chip at concentrations of 0.1–0.6 mg/ml (5–28 μm) and over the His-402 CM4 chip at concentrations of 0.1–0.5 mg/ml (5–22 μm). For the SCR-6/8 analyses, the CM4 chips were used with 0.3 mg/ml nonspecific binding reducer (GE Healthcare, Uppsala, Sweden) added to each sample to reduce nonspecific binding to dextran surfaces. Regeneration after each run was achieved by pulsing with 10 mm sodium acetate buffer, 5 m NaCl, 0.05 mm EDTA, pH 7.0, across both flow cells twice for 45 s. The maximum binding response value in each run was fitted to a steady-state 1:1 affinity model.

### X-ray scattering data

SAXS data were acquired in two sessions on Instrument ID02 (86) at the European Synchrotron Radiation Facility (Grenoble, France) with a ring energy of 6.0 GeV and operating in four-bunch mode. Storage ring currents were 30–44 and 68–89 mA, and the sample-detector distances were 2 and 3 m, respectively. Radiation damage was eliminated by the continuous movement of the sample in its capillary flow cell during beam exposure, the use of 10 time frames of duration between 0.1 and 0.5 s each during each acquisition, and on-line checks for the absence of radiation damage at low *Q*. Two FH Tyr-402 and two FH His-402 allotypes were each studied at five concentrations between 0.4 mg/ml (2.6 μm) and 3.3 mg/ml (21.4 μm). Measurements were done in HEPES buffer. Other details including data reduction are described elsewhere (30, 31).

In a given solute-solvent contrast, the radius of gyration (*R_g_*) is a measure of structural elongation if the internal inhomogeneity of scattering densities within the protein has no effect. Guinier analyses at low *Q* gives the *R_g_*, and the forward scattering at zero angle *I*(0) (87) as shown in Equation 1,

(Eq. 1) $$\ln I\left(Q\right)=\ln I\left(0\right)-R_{\text{G}}^{2}Q^{2}/3$$

This expression is valid in a *Q*·*R_g_* range up to 1.5. If the structure is elongated, the mean radius of gyration of cross-sectional structure *R_XS_* and the mean cross-sectional intensity at zero angle (*I*(*Q*)Q)*_Q_*_→ 0_ are obtained from Equation 2,

(Eq. 2) $$\ln\left(I\left(Q\right)Q\right)={\left(I\left(Q\right)Q\right)}_{Q\to 0}-R_{XS}^{2}Q^{2}/2$$

The *R_g_* and *R_XS_* parameters were calculated using the SCT suite of programs (88). Indirect transformation of the scattering data *I*(*Q*) in reciprocal space into real space to give the distance distribution function *P*(*r*) was carried out using the program GNOM: (89)

(Eq. 3) $$P\left(r\right)=\frac{1}{2\pi^{2}}\int_{o}^{\infty }I\left(Q\right)Qr\;\sin\left(Qr\right)dQ$$

*P*(*r*) corresponds to the distribution of distances *r* between volume elements. For this, the X-ray *I*(*Q*) curve utilized up to 207 data points for *Q* between 0.06 and 1.50 nm^−1^ for 0.4 mg/ml FH, and increasing up to 308 data points for *Q* between 0.06 and 2.10 nm^−1^ for 3.3 mg/ml FH. Other details are described elsewhere (29–31, 50).

### Atomistic FH scattering modeling

The first scattering modeling of the 20 SCR domains in FH used NMR structures for three SCR domains and homology models for the remaining 17 SCR domains (Fig. 1) (50). The second modeling used NMR and crystal structures for 11–14 SCR domains, and homology models for the remaining 9 SCR domains (26, 51). Here, a combination of MODELLER version 9.14 (90) and monomer Monte Carlo (SASSIE-web) (91) was used to build a starting FH model from NMR and crystal structures for 17 SCR domains and three improved SCR homology models for SCR-9, SCR-14, and SCR-17 (Tables 1 and 2). For the homology modeling, a template SCR structure without linker residues was identified using the basic local alignment search tool to search the Protein Data Bank server (BLAST-PDB) (Table 2) (92), and its sequence was aligned to the template sequence using Clustal Omega multiple sequence alignment (93). By this, low sequence identity was mostly confined to the loop regions, and the secondary structure was conserved. These loop regions were modeled using the loop optimization protocol in MODELLER. The best SCR model was selected from a final dataset of 100 generated models using the normalized discrete optimized protein energy (DOPE) score (94). For the SCR-9, SCR-14, and SCR-17 homology models and the NMR-based SCR-5 model (95), strained high-energy bonds were relaxed to give physically stable models by energy minimization (500,000 steps) and molecular dynamics simulations (1,000,000 steps) in the simulation engine NAMD version 2.9 (96). The PDB Reader input generator tool (97, 98) converted the PDB file into CHARMM readable format, generated a protein structure file (PSF), including the disulfide bonds, and provided the CHARMM36 force-field files (99, 100). The quality of the secondary structure was verified using Ramachandran plots on the RAMPAGE server (101), the DSSP program (102, 103), and visual comparison with other SCR domains. As required, the Ramachandran plots showed a minimal number of outliers in these four SCR models. To create the initial full-length FH structure (Fig. 1), the 11 SCR fragment models (SCR-1/4, SCR-5, SCR-6/8(His-402), SCR-9, SCR-10/11, SCR-11/12, SCR-12/13, SCR-14, SCR-15/16, SCR-17, and SCR-18/20) were aligned to the FH sequence sequentially using MODELLER. Each of SCR-1/5, SCR-6/13, SCR-14/16, and SCR-17/20 were assembled first, before their simultaneous alignment to the full-length FH sequence. The best FH model was selected based on the normalized DOPE score and then refined in NAMD to give the final FH initial structure. FH was numbered from the N terminus of the 18-residue signal peptide to follow HGVS convention. Eight biantennary disialylated glycans (18) were added to Asn-511 in SCR-9, Asn-700 in SCR-12, Asn-784 in SCR-13, Asn-804 in SCR-14, Asn-864 and Asn-892 in SCR-15, Asn-1011 in SCR-17, and Asn-1077 in SCR-19 (Fig. 1). For each glycan–Asn Protein Data Bank (PDB) file, GlycanReader was used to generate the CHARMM force field and PSF inputs for energy minimization in NAMD. The resulting energy-minimized glycans were positioned onto FH by superimposing the Asn residues in PyMOL and then deleting the Asn residue in the glycan PDB. Once all eight glycans had been added to FH and accepted by GlycanReader, bash scripts were used to convert the nomenclature and numbering of the glycan and protein atoms to the format required for the torsion angle Monte Carlo (TAMC) module in SASSIE-web. In this, the glycans were moved with their attached protein segment and not varied independently.

The model library of physically realistic FH structural conformations was generated by subjecting the inter-SCR linkers to the TAMC module in SASSIE-web (104). Eleven of the 19 linkers were defined by high-resolution structures and were therefore held fixed. The remaining eight linkers at SCR-4/5, SCR-5/6, SCR-8/9, SCR-9/10, SCR-13/14, SCR-14/15, SCR-16/17, and SCR-17/18 were moved (*arrowed,* Fig. 1). For each of these linker residues, the backbone ϕ and ψ torsion angles were varied in steps of up to either 30 or 180°, except for the torsion angles involving the conserved Cys residues at the start and end of each linker. To maximize the sampling, four simulations were run as follows: (i) 200,000 steps with up to 30° moves using the FH initial model; (ii) 200,000 steps with up to 180° moves using the FH initial model; (iii) 100,000 steps with up to 30° moves using an FH model from simulations i and ii with its CT bent inwards, and (iv) 10,000 steps with up to 30° moves using the best-fit FH model from simulations i–iii. In a Monte Carlo simulation, many moves result in structures with steric clashes and were discarded as physically unrealistic. In the present case, the 510,000 attempted moves resulted in 15,136 (8%), 3720 (1%), 10,316 (10%), and 543 (5%) physically-realistic, acceptable models, respectively. These were combined into a library of 29,715 models for SAXS curve fitting. A theoretical scattering curve was generated from each model using SasCalc. SasCalc calculates the scattering curve using an exact all-atom expression for the scattering intensity in which the orientations of the *Q* vectors are taken from a quasi-uniform spherical grid generated by the golden ratio (105).

For each of the Tyr-402 and His-402 allotypes, the experimental scattering curve with 221 data points was extrapolated to zero concentration to eliminate the contribution of self-association. These were compared with the theoretical curves using the *R*-factor shown in Equation 4,

(Eq. 4) $$R=\sum_{Q_{i}}\frac{\left|\left(I_{\exp}\left(Q_{i}\right)-I_{\text{model}}\left(Q_{i}\right)\right)\right|}{\left|\left(I_{\exp}\left(Q_{i}\right)\right)\right|}$$

where *Q_i_* is the *Q* value of the *i*th data point; *I*_exp_(*Q_i_*) is the experimental scattering intensity; and *I*_model_(*Q_i_*) is the theoretical modeled scattering intensity (88). For SasCalc, the lowest *Q* values before the Guinier *R_g_* region in the extrapolated scattering curves were interpolated to zero *Q* using MATLAB. After interpolation, the original 221 *I*(*Q*) values between *Q* of 0.0 and 1.5 nm^−1^ were retained to define the *Q* spacing for SasCalc. The *R*-factor *versus R_g_* graphs were similar for the extrapolated and 0.4 mg/ml curves, whereas those from curve fitting at 0.7, 1.1, and 2.2 mg/ml gave worse *R*-factors (data not shown), as expected. For each of the four Tyr-402 and His-402 curves, the 29,715 models were filtered on both *R_g_* and *R*-factor. Models were accepted if their *R_g_* values were within ±5% of the experimental *R_g_* values (Table 3), and their *R*-factor was ≤5%. The best-fit 100 models were identified by ranking the filtered models by their *R*-factors. The Y402H polymorphism had no effect on the curve fits, leading to an *R*-factor difference of only 0.0003%.

The SCR domain arrangement in the 29,715 FH models was parameterized using the separations between the FH COM and the NT and CT α-carbon atoms. Separations were calculated using Tcl scripting within Visual Molecular Dynamics (VMD) software (106), and their frequencies were displayed as histograms using R (107) for statistical analyses. To assess the normality of the separation distributions, Quantile-Quantile plots and two-tailed Kolmorogov-Smirnov tests were employed using the ks.test statistical function in R (107, 108). Here, the null hypothesis stated that the distribution was consistent with the normal distribution, and a Bonferroni-corrected significance level of 0.05 divided by 2 (0.025) was set. The Kolmorogov-Smirnov test calculated the maximum vertical deviation between the two curves as the test statistic *D* and the probability of *D* occurring due to chance (*p* value). To compare the distributions for all 29,715 models, the 310–2678 filtered models, and the best-fit 100 models of the two allotypes, their distributions were converted to cumulative density frequencies and compared using the two-tailed Kolmorogov-Smirnov test in R with the ks.test statistical function. Here, for each comparison, the null hypothesis stated that the distributions were the same and a Bonferroni-corrected significance level of 0.05 divided by 12 (0.00417) was set. To visualize the resulting conformations after applying the *R_g_* and *R*-factor filters, the filtered models were superimposed with the reference FH model using SCR-10/13, and visualized as density plots using SASSIE-web.

Principal component analysis (PCA) provided by the Bio3d package in R (108) identified four major conformational states in the best-fit 100 models. For both allotypes, PCA satisfactorily accounted for >80% of the variance between the models. The average FH structure for each PCA group was identified using a centroid model computed using R. The SCR domain arrangement of each PCA group was identified by a density plot using SASSIE-web. To assess the conformational differences between the two allotypes, the frequencies of the two major SCR domain arrangements were compared by using the two-tailed Fisher's test for a 2 × 2 contingency table in GraphPad QuickCalcs (https://www.graphpad.com/quickcalcs/).^4^ Here, the null hypothesis stated that the proportion of FH models with an inwardly-bent NT and extended CT, compared with an extended NT and inwardly-bent CT, were the same. The significance level was set as 0.05, and the probability of the difference occurring due to chance (*p* value) was computed. To see whether these two conformations could be distinguished by their SAXS curves, the difference in scattering intensity between their best fitting centroid model curves was calculated and visualized using R. The FH models were assessed by superimposing the centroid model for each of the largest ensembles with crystal structures for the FH-ligand complexes of SCR-1/4 with C3b and SCR-19/20 with C3d using PyMOL (PDB codes 2WII and 5NBQ) (52, 78).

For the AUC modeling, the theoretical *s*_20, *w*_^0^ values for the FH models were calculated directly from the atomic coordinates with the default value of 0.31 nm for the atomic element radius for all atoms to represent the hydration shell by using the HYDROPRO shell modeling program (109). The molecular weight and partial specific volume for FH used followed that used previously (26, 110). FH glycosylation was considered by using the compiled partial specific volumes for saccharides (111).

### Links to web servers and tools

The following were used: BLAST (https://blast.ncbi.nlm.nih.gov/Blast.cgi)^4^; Clustal Omega (http://www.ebi.ac.uk/Tools/msa/clustalo/)^4^; SASSIE-web (https://sassie-web.chem.utk.edu/sassie2/)^4^; CHARMM-GUI Glycan Reader (http://charmm-gui.org/?doc=input/glycan)^4^; CHARMM-GUI PDB Reader (http://www.charmm-gui.org/?doc=input/pdbreader)^4^; RAMPAGE (http://mordred.bioc.cam.ac.uk/∼rapper/)^4^; DSSP (http://swift.cmbi.ru.nl/gv/dssp/)^4^; and GraphPad QuickCalcs (https://www.graphpad.com/quickcalcs/contingency1/).^4^

## Author contributions

A. J. O., R. N., and S. J. P. conceptualization; S. J. P. supervision; A. J. O., R. N., A. M., J. G., and S. J. P. investigation; A. J. O., R. N., A. M., J. S. B., and J. G. methodology; A. J. O., R. N., and S. J. P. writing-original draft; A. J. O., R. N., and S. J. P. writing-review and editing; R. N. and S. J. P. formal analysis; J. S. B. software; S. J. P. funding acquisition.

## Supplementary Material

Supporting Information

## Footnotes

4 Please note that the JBC is not responsible for the long-term archiving and maintenance of this site or any other third party hosted site.

## Footnotes

3 The abbreviations used are:

FH: factor H
AMD: age-related macular degeneration
SCR: short complement regulator
SPR: surface plasmon resonance
SAXS: small-angle X-ray scattering
FI: factor
NT: N-terminal
CT: C-terminal
COM: center of mass
AUC: analytical ultracentrifugation
PDB: Protein Data Bank
PSF: protein structure file
RU: response unit
TAMC: torsion angle Monte Carlo
CRP: C-reactive protein
HS: heparan sulfate
PCA: principal component analysis
DOPE: discrete optimized protein energy
aHUS: atypical hemolytic uremic syndrome
C3G: C3 glomerulopathy
HSA: human serum albumin.
