# Supplementary material for: Two distinct conformations of factor H regulate discrete complement-binding functions in the fluid phase and at cell surfaces
Source: J Biol Chem. 2018 Sep 14;293(44):17166–87. doi: 10.1074/jbc.RA118.004767 (PMC6222095; doi:10.1074/jbc.RA118.004767)
Supplement: Supporting Information [file supp_293_44_17166__index.html]

Two distinct conformations of factor H regulate discrete complement-binding functions in the fluid phase and at cell surfaces — Solution structures of FH Tyr402 and His402 — Two distinct conformations of factor H regulate discrete complement-binding functions in the fluid phase and at cell surfaces — Solution structures of FH Tyr-402 and His-402 — Supporting Information 

# Two distinct conformations of factor H regulate discrete complement-binding functions in the fluid phase and at cell surfaces

## Supporting Information

- Supporting Information (to be published online) - FH His402 PDB file for Fig 14b
- Supporting Information (to be published online) - FH Tyr402 PDB file for Fig 14a
- Supporting Information (to be published online) - DCD files for the 100 best fit models for the Tyr and His allotypes.
